# Rapid syphilis testing among young people: prevalence and associated factors*

**DOI:** 10.1590/1518-8345.7488.4958

**Published:** 2026-10-07

**Authors:** William Caracas Moreira, Leidyanny Barbosa de Medeiros, Rute Xavier Silva, Jordana de Almeida Nogueira, Mônica Oliveira Batista Oriá, Oriana Deyze Correia Paiva Leadebal

**Affiliations:** 1 Universidade Federal do Ceará, Campos Porangabuçu, Fortaleza, CE, Brazil.; 2 Universidade Federal da Paraíba, Campus I, João Pessoa, PB, Brazil.; 3 Scholarship holder at the Coordenação de Aperfeiçoamento de Pessoal de Nível Superior (CAPES), Brazil.; 4 Scholarship holder at the Conselho Nacional de Desenvolvimento Científico e Tecnológico (CNPq), Brazil.

**Keywords:** Syphilis, Young Adult, Sexual Behavior, Sexual Vulnerability, Sexually Transmitted Diseases, Prevalence

## Abstract

**Objective:**

to analyze the prevalence of rapid syphilis testing among young people and its associated factors.

**Method:**

cross-sectional study, conducted in two Testing and Counseling Centers. It involved 301 young people, whose information was collected using a validated instrument and analyzed using descriptive statistics, chi-square tests, prevalence ratios, and logistic regression.

**Results:**

young people had a prevalence of 14.9% for rapid syphilis testing, with significant associations with sociodemographic factors (male gender, transgender identity, and non-heterosexual sexual orientation), socioeconomic factors (low income, independent living, and history of institutionalization), and behavioral factors (transactional sex, risky sexual practices, history of sexually transmitted infections, and drug use). Multivariate analysis confirmed higher odds of rapid syphilis testing among low-income, male youth with a history of sexually transmitted infections, reinforcing the vulnerability of these groups.

**Conclusion:**

In summary, the prevalence and factors associated with rapid syphilis testing among young people permeate an intersectional structure with multiple layers of vulnerabilities, encompassing sociodemographic, sociostructural, and behavioral dimensions.

## Highlights

**(1)** The prevalence of rapid syphilis testing among young people was estimated at 14.9%.

**(2)** Male gender and previous STIs increase the risk of testing positive for syphilis in rapid tests.

**(3)** Socio-structural factors increase vulnerability to testing positive for syphilis in rapid tests.

**(4)** Risky sexual practices were associated with testing positive for syphilis in rapid tests.

**(5)** Low income and discrimination increase the chance of testing positive for syphilis in rapid tests.

## Introduction

Syphilis is a sexually transmitted infection (STI) that must be reported, caused by *Treponema pallidum* and transmitted: from mother to fetus during pregnancy; at the time of delivery, through the bloodstream; or through sexual intercourse without proper use of a condom^1^. With diagnosis available through the Unified Health System (SUS), the infection can be quickly detected and treated to prevent clinical progression, the emergence of complications, an increase in cases of congenital syphilis, and new cases of acquired infection^2^. Even so, recent studies highlight the reemerging nature of the disease and a significant number of cases among young people, especially those under the age of 24^1^ ^)-(^ ^3^.

With regard to early diagnosis strategies, there is rapid testing (RT) for syphilis^4^, which consists of an immunochromatographic method capable of detecting antibodies against Treponema pallidum from small blood samples, preferably obtained by finger prick, which promotes speed and ease of collection, even in environments with poor working infrastructure^4^ ^)-(^ ^5^. The result, available in a few minutes, can be classified as reactive or non-reactive for syphilis. A non-reactive result implies reinforcement of preventive measures and possible repetition of RT in 30 days, if there is clinical suspicion or recent risk. On the other hand, reactive results require confirmation by a non-treponemal test, such as the Venereal Disease Research Laboratory (VDRL) test, in order to distinguish active infection from serological scarring^5^.

Comprising adolescence and young adulthood, youth occurs between the ages of 15 and 24, a period in which people experience discoveries in different aspects, which in turn shape social relationships and contribute to behaviors, including sexual behaviors^6^. From this perspective, studies emphasize that people’s sexual expression has been emerging earlier and earlier and is commonly associated with deficits in knowledge about STIs and difficulties for parents and health professionals in addressing the issue in the population, according to the conditions of vulnerability to syphilis among young people^6^ ^)-(^ ^7^.

Globally, the Global Burden of Disease study showed that, although syphilis is not the most prevalent STI in absolute numbers, its age-standardized rate showed continuous growth between 1990 and 2021, with Brazil standing out as the country with the highest increase in this rate among the countries analyzed^8^.

In this context, it is noted that between 2011 and 2022, Brazil reported more than 1.1 million cases of acquired syphilis, of which 36% occurred in young people. Between 2020 and 2021, the national detection rate rose from 59.1 to 78.5 cases per 100,000 inhabitants, a trend also observed in the Northeast region, where rates jumped from 29.9 to 47.3 cases per 100,000 inhabitants. Paraíba, a state in the Northeast, showed growth similar to the regional pattern, although without exceeding the national average^2^. In addition, it is worth noting that the data on mandatory notifications for syphilis in Paraíba follow the global trend^9^, i.e., they have decreased since the beginning of the COVID-19 pandemic, which certainly does not reflect a reduction in the occurrence of infection, but rather fragility in diagnosis, underreporting, and, consequently, lack of timely treatment^10^.

This study, in addition to highlighting the importance of RT for syphilis in the context of public health^5^, emphasizes the vulnerability of a specific age group (young people aged 15 to 24) to syphilis, differing from other studies that mainly address general populations, pregnant women, or key groups linked primarily to gender identity and/or sexual orientation, according to a systematic review of the literature^11^. 

Combined with the scientific need to contribute to the Sustainable Development Goals (SDGs) of the 2030 Agenda of the United Nations (UN)^12^, the production of studies on the prevalence of acquired syphilis among young people finds solid justification both in the global and Brazilian epidemiological scenario^2^ ^),(^ ^8^ and in the dynamics of health research^7^. 

Given the above, the objective of this study was to analyze the prevalence of RT reactive for syphilis among young people and its associated factors.

## Method

### Study type or design

Cross-sectional study, developed from an excerpt of the primary research entitled “HIV in the young population: subsidies for tackling the epidemic based on the analysis of socio-structural and behavioral factors”, reported according to the recommendations of Strengthening the Reporting of OBservational studies in Epidemiology (STROBE)^13^_._

### Study location

Developed in two Testing and Counseling Centers in two municipalities in health macro-regions I and II, in the state of Paraíba, Brazil. These services operate on a walk-in basis or by referral from other services, in the morning and afternoon shifts, serving 134 of the state’s 223 municipalities and offering sexual health promotion, prevention, diagnosis, and treatment services. 

### Period

Data collection took place from February to July 2021.

### Population

The population consisted of young people aged between 15 and 24 years old. 

### Sample definition

The sample was defined by calculation for finite populations, considering the average monthly estimate of RT performed in the services investigated (Macro-region I Headquarters - N=500; Macro-region II Headquarters - N=320); significance level of 5%; confidence interval 95%CI; and an anticipated Prevalence (P) value based on the detection rate of HIV/AIDS cases in the recommended age group, focusing on the original research (Macro-region I Headquarters with P = 23.0%; Macro-region I Headquarters with P = 8.6%); which defined the sample as 301 young people, of whom 200 are from Macro-region I Headquarters and 101 from Macro-region II Headquarters.

### Selection criteria

Participants were selected using non-probabilistic sampling, adopting convenience criteria based on the specific characteristics of the study site. People whose serological status for syphilis was unknown at the time of the primary survey and who sought out Testing and Counseling Centers in order to undergo RT for any STI were included. In addition, those who had a disability that prevented them from responding to the questionnaire were excluded.

### Instrument used and study variables

The instrument was developed and validated by researchers from the Center for Studies on HIV, Health, and Sexuality (NEHAS)^14^. It consists of 45 questions divided into four sections: 1 - Interview identification; 2 - Sociodemographic variables (age, gender, aspects related to sexuality, self-declared race, years of schooling); 3 - Socio-structural variables (work, current relationship status, housing conditions, family income - the minimum wage of R$ 1,100.00 (in force in 2021) was considered, history of institutionalization, discrimination, psychiatric treatment, and victimization by sexual violence); and 4 - Behavioral variables (*coitarca*, history of STIs, consent in sexual relations, aspects related to sexual practices, transactional sex, drug use)^14^.

### Data collection

Data collection took place during morning and afternoon shifts, according to the availability of the researchers, varying according to the days of the week, until the desired sample was obtained. 

As established in the inclusion and exclusion criteria, young people who sought out the Testing and Counseling Centers were informed about the study and invited to participate. Acceptance required the signing of the Free and Informed Consent Form or the Free and Informed Assent Form for minors under 18 years of age. Consequently, they participated in individualized interviews in a private setting, guided by the data collection instrument^14^ with a face-to-face approach by a researcher trained to conduct it. It should be noted that this process preceded the participant’s RT.

### Data processing and analysis

The data of interest in this study were transferred from the original research database and organized in a Microsoft Excel^®^ spreadsheet for compilation and recategorization using the Statistical Package for the Social Sciences (SPSS) version 20.0. 

To analyze the characteristics of the participants, absolute and relative frequencies were calculated, as well as measures of central tendency and dispersion. 

To analyze prevalence, the number of RT reagents for syphilis was divided by the total sample (n= 301). To calculate Pearson’s chi-square (X²) and prevalence ratio (PR), the association between the outcome RT reactive for syphilis and the associated factors was considered. It should be noted that for these analyses, data referring to the categories “ignored”, “did not want to answer”, and “did not know how to answer” were excluded.

For the multivariate analysis, we chose to use the logistic regression model, adopting backward selection to select the variables that best explained the phenomenon under study, considering a significance level of 5% (p < 0.05)^15^.

### Ethical aspects

This study followed the ethical precepts for research involving human subjects set forth in Resolution No. 466/12 of the National Health Council; it was approved by the Research Ethics Committee of the Health Sciences Center of the Federal University of Paraíba - Opinion No. 3,935,713 (03/26/2020), and the necessary procedures were conducted to ensure the protection of information accessible to the research team and anonymity at the time of publication of the results.

## Results

Of the 382 young people approached by researchers during data collection, 301 agreed to participate. The participants had a mean age of 20.83 years (standard deviation ± 2.3), whose sociodemographic characterization indicated a predominance of the 20-24 age group (212/70.4%), male gender (155/51.5%); self-declared non-white skin color white (217/72.1%); cisgender (291/96.6%), heterosexual (175/58.1%), with more than eight years of schooling (259/86%), and 86% (163) were not currently studying. 

Regarding factors intrinsic to the socio-structural dimension, there was a predominance of young people in stable relationships (172/57.3%), with family incomes between one and two minimum wages (111/37.2%), who do not work (166/55.1%) and share housing only with family members (152/50.5%). They also reported victimization due to discrimination based on sexual orientation (104/34.7%) and gender identity (57/18.9%), mainly in schools and universities (86/82.7% and 11/19.6%, respectively). In addition, sexual violence was reported (84/27.9%) - perpetrated mainly by strangers (29/35%), history of psychiatric treatment (61/20.3%), and imprisonment or institutionalization (10/3.3%).

In the behavioral profile of young people, the following characteristics were observed to be predominant: sexual intercourse before the age of 15 (153/50.8%) - a practice not consented to by a portion of young people (15/5%); condom use “sometimes” (167/55.4%); practicing oral sex (237/78.7%) and vaginal sex (214/71.1%); and alcohol consumption (174/57.8%), of which a portion (68/39.5%) reported having stopped using condoms when under the influence of alcohol; another stated that they did not remember whether or not they used condoms on such occasions (11/6.4%).

There were reports of transactional sex (29/9.6%), insertive anal sex (114/37.9%) and receptive anal sex (124/41.2%), previous diagnosis of STIs (63/20.9%), difficulty in acquiring (32/10.6%) or negotiating condoms (103/34.2%), and illicit drug use was also reported (103/34.2%).

Regarding prevalence and associations inherent to the results, a prevalence of RT reactive for syphilis was identified in a portion of young people (45/14.9%) and an association with sociodemographic, sociostructural, and behavioral factors.

Regarding sociodemographic factors, the outcome for syphilis-reactive RT was associated with variables related to sex, gender identity, and sexual orientation (Table 1). In this dimension (Table 1), males had an OR of 3.8 (95% CI: 1.9-7.5) times higher for RT reactive for syphilis, while young people whose gender identity was trans male or trans female had PRs of 8.0 (95% CI: 1.7-36.9) and 10.1 (95% CI: 4.4-23.1), respectively. Furthermore, being homosexual or pansexual showed, respectively, an OR of 4.4 (95% CI: 2.3-8.4) and 4.8 (95% CI: 1.6-14.2), indicating greater vulnerability to syphilis-reactive RT in these groups.

**Table 1:** Prevalence (P*), prevalence ratio (PR^†^), and chi-square test of association between the outcome of rapid syphilis testing and sociodemographic factors among young people (n = 301). João Pessoa, PB, Brazil, 2021

Variables		Rapid syphilis test reagent (N)		P* (%)	PR^†^ [95.0%CI^‡^]	**X² *p*-value** ^§^
		Yes	No			
Age group	15-19 years	08	81	9.0	1	0.060
	20-24 years	37	175	17.5	1.9 [0.9; 4.0]	
Sex	Female	09	137	6.2	1	0.001^||^
	Male	36	119	23.2	3.8 [1.9; 7.5]	
Self-declared color	White	12	72	14.3	1	0.841
	Not white	33	184	15.2	1.00 [0.6; 1.9]	
Years of study	≤ 8 years	04	38	9.5	1	0.288
	> 8 years	41	218	15.8	1.7 [0.6; 4.4]	
Study	Yes	19	119	13.8	1	0.597
	No	26	137	16.0	1.2 [0.6; 2.0]	
Gender identity	Man	30	116	20.5	3.3 [1.6; 6.7]	0.001^||^
	Woman	09	136	6.2	1	
	Trans man	01	1	50.0	8.0 [1.7; 36.9]	
	Trans woman	05	3	62.5	10.1 [4.4; 23.1]	
Sexual orientation	Heterosexual	12	163	6.9	1	0.001^||^
	Homosexual	24	55	30.4	4.4 [2.3; 8.4]	
	Bisexual	06	32	15.8	2.0 [0.9; 5.7]	
	Pansexual	03	6	33.3	4.8 [1.6; 14.2]	

*P = Prevalence; ^†^PR = Prevalence ratio; ^‡^CI = Confidence interval; ^§^X² p value = p value of the Chi-square test for proportion comparison; ^||^p-value <0.05 = Association between rapid syphilis testing and the corresponding variable

In terms of socioeconomic factors, a statistical association was found between RT reactivity for syphilis and the following variables: family income, with whom they share their home, victimization due to discrimination based on gender identity and sexual orientation, and having been arrested or institutionalized. Thus, it should be noted that a household income below the minimum wage increased the OR to 2.3 (95% CI: 1.2-4.6) times for syphilis-reactive RT. In addition, living only with friends or alone presented, respectively, PR 3.9 (95% CI: 1.9-8.0) and 3.2 (95% CI: 1.6-6.3). A similar phenomenon occurs with young people who had a history of imprisonment/institutionalization, with an OR of 2.8 (95% CI: 1.7-6.4) (Table 2).

**Table 2:** Prevalence (P*), prevalence ratio (PR^†^), and chi-square test of association between the outcome of rapid syphilis testing and socioeconomic factors among young people (n = 301). João Pessoa, PB, Brazil, 2021

Variables		Rapid syphilis test reagent (N)		P* (%)	PR^†^ [95.0%CI^‡^]	**X² *p* value** ^§^
		Yes	No			
Household income	≤ 1 MW^||^	23	70	24.7	2.3 [1.2; 4.6]	0.008^¶^
	1 to 2 MW^||^	12	99	10.8	1.0 [0.5; 2.2]	
	> 2 MW^||^	10	84	10.6	1	
Works	Yes	20	115	14.8	1	0.953
	No	25	141	15.1	1.0 [0.6; 1.7]	
Who do you share your home with?	Only with friends	08	13	38.1	3.9 [1.9; 8.0]	0.001^¶^
	Alone	12	26	31.6	3.2 [1.6; 6.3]	
	Only with partner	07	37	15.9	1.6 [0.7; 3.7]	
	Only with family members	15	137	9.9	1	
	Partner and friends or family members	03	43	6.5	0.7 [0.2; 2.2]	
Relationship status	Fixed	17	155	9.9	1	0.051
	Casual	11	33	25.0	2.5 [1.3; 5.0]	
	With more than one partnership	02	12	14.3	1.4 [0.3; 5.6]	
	No relationship	15	55	21.4	2.1 [1.1; 4.1]	
Discrimination based on gender identity	Yes	15	42	26.3	2.1 [1.2; 3.7]	0.008^¶^
	No	30	214	12.3	1	
Discrimination based on sexual orientation	Yes	29	75	27.9	3.4 [1.9; 6.0]	0.001^¶^
	No	16	179	8.2	1	
Victimization due to sexual violence	Yes	16	68	19.0	1.4 [0.8; 2.5]	0.215
	No	29	188	13.4	1	
Has been incarcerated or institutionalized	Yes	04	6	40.0	2.8 [1.7; 6.4]	0.024^¶^
	No	41	250	14.1	1	
History of psychiatric treatment	Yes	11	50	18.0	1.3 [0.7; 2.3]	0.450
	No	34	206	14.2	1	

*P = Prevalence; ^†^PR = Prevalence ratio; ^‡^CI = Confidence interval; ^§^X² p value = p value of the Chi-square test for proportion comparison; ^||^MW = Minimum wage of R$ 1,100.00, Brazil, effective in 2021; ^¶^p-value <0.05 = Association between rapid syphilis testing and the corresponding variable

Regarding behavioral factors, syphilis-reactive RT was associated with non-consent during first sexual intercourse, condom use frequency, transactional sex, vaginal sex, insertive and receptive anal sex, history of STIs, and illicit drug use (Table 3). Thus, it was observed that non-consensual first sexual intercourse increased the OR by 2.3 (95% CI: 1.1-5.1) for syphilis-reactive RT, as did the following sexual practices: transactional sex increased the OR by 3.8 (95% CI: 2.2-6.4), insertive anal sex increased the OR by 2.6 (95% CI: 1.5-4.6), and receptive anal sex increased the OR by 1.7 (95% CI: 1.1-3.0). In addition, young people who did not engage in vaginal sex had an OR 3.8 (95% CI: 2.2-6.6) times higher for syphilis-reactive RT (Table 3).

**Table 3:** Prevalence (P*), prevalence ratio (PR^†^), and chi-square test of association between the outcome of rapid syphilis testing and behavioral factors among young people (n = 301). João Pessoa, PB, Brazil, 2021

Variables		Rapid syphilis test reagent (N)		P* (%)	PR^†^ [95.0%CI^‡^]	**X² *p*-value** ^§^
		Yes	No			
*Coitarca* (n= 294)	≤ 15 years	25	128	16.3	1.1 [0.6; 2.0]	0.608
	> 15 years	20	121	14.2	1	
First consensual sexual intercourse (n= 298)	Yes	40	243	14.1	1	0.043^||^
	No	05	10	33.3	2.3 [1.1; 5.1]	
Difficulty in obtaining condoms (n= 299)	Yes	08	24	25.0	1.8 [0.9; 3.5]	0.096
	No	37	230	13.9	1	
Frequency of condom use (n= 297)	Always	21	40	34.4	5.9 [2.1; 16.3]	0.001^||^
	Sometimes	20	147	12.0	2.0 [0.7; 5.8]	
	Never	04	65	5.8	1	
Difficulty negotiating condom use (n= 294)	Always	03	28	9.7	1	0.265
	Sometimes	15	57	20.8	2.1 [0.6; 6.9]	
	Never	27	164	14.1	1.4 [0.4; 4.5]	
Transactional sex	Yes	13	16	44.8	3.8 [2.2; 6.4]	0.001^||^
	No	32	240	11.8	1	
Practices vaginal sex (n=297)	Yes	18	196	8.4	1	0.001^||^
	No	27	56	32.5	3.8 [2.2; 6.6]	
Practice oral sex (n=297)	Yes	38	199	16	1.3 [0.6; 2.9]	0.399
	No	7	53	11.7	1	
Practicing insertive anal sex (n=297)	Yes	28	86	24.6	2.6 [1.5; 4.6]	0.001^||^
	No	17	166	9.3	1	
Practicing receptive anal sex (n=297)	Yes	25	99	20.2	1.7 [1.1; 3.0]	0.041^||^
	No	20	153	11.6	1	
History of STIs^¶^ (n= 289)	Yes	30	33	47.6	7.1 [4.1; 12.5]	0.001^||^
	No	15	211	6.6	1	
Consumes alcoholic beverages	Yes	31	143	17.8	1.6 [0.9; 2.9]	0.103
	No	14	113	11.0	1	
Consumes illicit drugs	Yes	23	80	22.3	2.0 [1.2; 3.4]	0.010^||^
	No	22	176	11.1	1	

*P = Prevalence; ^†^PR = Prevalence ratio; ^‡^CI = Confidence interval; ^§^X² p value = p value of the chi-square test for proportion comparison; ^||^p-value <0.05 = Association between rapid syphilis testing and the corresponding variable; ^¶^STI = Sexually transmitted infections

In this context, a previous history of STIs and illicit drug use increased the OR by 7.1 (95% CI: 4.1-12.5) and 2.0 (95% CI: 1.2-3.4), respectively, for RT reactive for syphilis. Regarding condom use frequency, those who reported always using condoms had an OR 5.9 (95%CI 2.1-16.3) times higher for syphilis-reactive RT compared to young people who reported using condoms sometimes or never (Table 3).

Based on logistic regression analysis, being young, aged 15 to 19 years, constitutes a protective effect against syphilis-reactive RT when compared to young people aged 20 to 24 years. However, those with an income below the minimum wage or between one and two minimum wages were 2.9 and 2.4 times more likely to be RT reactive for syphilis, respectively, compared to young people with an income above three minimum wages. Similarly, being male increased the chances of RT reactive for syphilis threefold compared to females, and having a history of STIs represented an 8.4 times greater chance of RT reactive for syphilis compared to those who did not report such a history (Table 4).

**Table 4:** Logistic regression model considering factors associated with rapid syphilis testing among young people (n = 301). João Pessoa, PB, Brazil, 2021

Variables	Coefficient	Standard error	Odds ratio	Confidence interval (95%)	p value*
Age group 15-19 years old	-0.78	0.34	0.45	[0.23 - 0.89]	0.02^†^
Male gender	1.11	0.43	3.06	[1.31 - 7.11]	0.01^†^
Family income ≥ 3 minimum wages^‡^					0.01^†^
Family income ≤ 1 minimum wage^‡^	1.09	0.42	2.98	[1.31 - 6.80]	0.01^†^
Family income between 1-2 minimum wages^‡^	0.90	0.46	2.47	[1.01 - 6.09]	0.04^†^
Previous diagnosis of STI^§^	2.13	0.36	8.46	[4.11 - 17.42]	0.01^†^
**R² (Nagelkerke)** ^||^					70.5%
**Hosmer-Lemeshow test (p-value)**					0.20

*p-value = p value of the logistic regression model; ^†^p<0.05 = Association between rapid syphilis testing and the factors investigated; ^‡^SM = Current minimum wage = R$ 1,100.00, Brazil, 2021; ^§^STI = Sexually Transmitted Infection; ^||^R² (Nagelkerke) = Measures the performance of the model based on the variables used

## Discussion

The sociodemographic profile presented in this study is similar to that found in the national^16^ ^)-(^ ^17^ and international^7^ ^),(^ ^18^ ^)-(^ ^23^ literature related to the prevalence of young males^16^ ^)-(^ ^19^ ^),(^ ^21^ ^)-(^ ^22^, cisgender^7^, heterosexual^7^ ^),(^ ^16^, aged 20 to 24 years^20^, non-white^16^ ^),(^ ^19^ ^),(^ ^23^, and with more than eight years of schooling in their samples^16^. The only variable that stood out was “currently studying”, as most studies focusing on young people tend to address the university setting, which consequently influenced the variable in question^24^.

Young males assume the greatest risk not only for syphilis-reactive RT but also for other STIs^21^ ^)-(^ ^22^. That said, vulnerability may be linked to behaviors such as seeking sexual partners through the internet, early age at first intercourse, higher frequency of sexual intercourse, low adoption of self-care practices, and preservation of sexual health, when compared to females^17^ ^)-(^ ^18^ ^),(^ ^21^ ^)-(^ ^22^.

Young people aged 20 to 24 are more vulnerable to reactive RT for syphilis and other STIs, especially females, due to the convergence of unfavorable sociodemographic and economic factors, transactional sexual practices, and inconsistent condom use^25^ ^)-(^ ^26^. In this scenario, persistent misinformation about sexual health and exposure to risky behaviors amplify this vulnerability, which is fundamental for access to adequate information and collective analysis of the factors that interfere in this process^27^.

Beyond individual factors, the bi- and multivariate analyses presented in this study suggest the vulnerability of population groups linked to the LGBTQIAPN+ community, as they point to a higher risk of syphilis-reactive RT in males, transsexuals, homosexuals, or pansexuals. This sociodemographic situation may be linked to misinformation caused by a cascade of events based on structural and historical prejudice experienced by this group, whose impacts are perceived in access to health services, the availability of resources and information to prevent risky sexual behavior, STIs, and promote sexual health^28^ ^)-(^ ^30^.

Furthermore, socio-structural variables reflect complex patterns of vulnerability in this population group, with emphasis on intersectionalities between economic conditions, situations of violence, and mental health^31^. These conditions are well established in the literature when viewed separately^32^, however, evidence of the current situation, with its intersectionalities, in prevalence studies linked to the risk of reactive RT for syphilis is still under-explored.

In this context, despite the potential for social transformation in educational environments in terms of respect and human dignity, schools and universities perpetuate a prejudiced structure, which creates spaces for the reproduction of LGBTphobic violence^29^ ^),(^ ^33^. Another factor intrinsic to the socio-structural vulnerability of these young people is linked to a history of deprivation of liberty, particularly relevant in peripheral youth populations, which affects how young people relate to each other (by engaging in risky sexual behaviors), their insertion into the labor market, and how they perceive access to health care^34^.

Furthermore, reports of sexual violence against adolescents and young people are present from large urban centers to rural communities^35^, where the predominance of unknown perpetrators, as pointed out in this study, contrasts partially with a study conducted in the interior of Piauí, which identified intimate partners and friends as the main perpetrators, suggesting that this is the reality of specific contexts of urban violence or underreporting of intra-family cases^36^.

Family income is a socio-structural factor inherent to access to health and information, since the higher the income, the better the access to health services and actions^37^. That said, studies show that the lower the family income, the greater the likelihood of inconsistent condom use and, consequently, the greater the risk for STIs^31^ ^),(^ ^37^ ^)-(^ ^38^.

As for the variables “not currently working” and “not currently studying”, the literature explains that young people experience typical aspects of emerging adulthood, permeated by social markers such as gender, education, and work experience, in addition to being affected by the precariousness of the labor market^23^. This condition, in addition to limiting opportunities for personal and social development, exposes these young people to multiple situations of vulnerability. Such factors are associated with contexts of greater social isolation, difficulty in accessing information and health services, and the adoption of risky behaviors, which makes this young population especially susceptible to STIs^23^.

In our sample, at least half of the young people reported living only with family members, which, based on the literature, constitutes a protective factor against STIs^39^ ^)-(^ ^40^. However, those who live with friends and/or alone represented a higher risk for syphilis-reactive RT and other STIs, since young people have greater freedom and little supervision, thus engaging in risky sexual behaviors^39^. Furthermore, it should be noted that the age group (15-19 years) understood as a protective factor against syphilis-reactive RT in this study can be explained by living with family members, high supervision, and late sexual debut^40^.

Regarding behavioral factors, it is observed that the relational context of young people determines behavioral patterns that directly influence the dynamics of sexual protection and creates different scenarios of epidemiological vulnerability^24^. However, the estimated risk of STIs based on the relationship status assumed by young people is not yet a scientific consensus. That said, behavioral analyses show that casual partners are more prone to prophylactic negligence-whether due to forgetfulness or deliberate disregard-while stable relationships favor the progressive replacement of condoms with interpersonal trust criteria^21^ ^),(^ ^24^ ^),(^ ^41^. In this sense, both models can increase exposure to syphilis and other STIs, as the reduction in consistent use of physical barriers facilitates transmission during sexual practices, regardless of subjective perception of safety^24^.

A review of the scope showed that sexual initiation among young people occurs, on average, at age 15^24^. In this context, the first sexual experience assumes epidemiological relevance, since the absence of condoms and/or lack of consent increase risky behaviors and vulnerability to STIs^21^ ^),(^ ^24^. Thus, unprotected or non-consensual intercourse is an important factor in the exposure and perpetuation of these infections among young people^21^ ^),(^ ^42^. 

With regard to the aforementioned dimension of knowledge, it is clear that coercion and sexual abuse are not only related to unwanted pregnancy, but also to the risk of STIs. In turn, the incidence of non-consensual sexual relations is an indicator of gender inequalities and prevailing patterns of masculinity^31^. Thus, it can be seen that coerced and forced sexual initiation is associated with inconsistent condom use, recent experiences of violence, and mental health outcomes among young people, a situation that highlights the multiple layers of vulnerability among young people, especially females^31^ ^),(^ ^42^. 

It is currently recognized that transactional sex translates into a factor of behavioral vulnerability, social, economic, and/or structural interfaces^21^ ^),(^ ^24^. In this sense, one study pointed out that street youth often neglect condom use during transactional sex due to pressure from intimate partners, immediate financial needs, and fear of violence, which describes a scenario conducive to the transmission of syphilis and other STIs^43^. 

The aforementioned dynamic is reproduced in different contexts: among South African university students, 1.4% of the 918 surveyed reported engaging in this practice^44^; while among American high school adolescents, the prevalence reached 7.4%, with males being 2.5 times more likely to engage in this practice^45^. Additionally, structural factors such as poverty, social exclusion, and limited access to health services perpetuate cycles of vulnerability and hinder both prevention and early diagnosis of these infections^24^ ^),(^ ^43^.

Among the sexual practices engaged in by young people, anal sex emerges as a factor of greater predisposition to reactive TR for syphilis and other STIs^24^, due to a combination of aspects such as: greater fragility of the rectal epithelium to infections; lower perception of risk, whose feeling of invulnerability enables the adoption of risky sexual behaviors; and deficits in access to adequate information and health services^46^.

It is well established in the literature that a history of STIs indicates previous exposure to risky behaviors, which tend to be repeated over time^21^ ^),(^ ^24^. Literature reviews demonstrate a significant association between a history of STIs and a higher risk of reactive RT for syphilis and other infections, corroborating the persistence of behavioral vulnerabilities^21^ ^),(^ ^24^. In this sense, the phenomenon is detailed by studies that have shown that people with a history of STIs are 1.8 times more likely to acquire other STIs^27^, while a previous diagnosis of two other STIs increases the probability of syphilis infection by 5.83 times^47^.

In another context, the association between alcohol and/or illicit drug use and unsafe sexual practices among young people is a critical factor in the transmission of syphilis and other STIs. One study found that the use of psychoactive substances alters judgment patterns and reduces risk perception during social interactions-at festive events, young people are less concerned about the subsequent risks and, consequently, engage in risky sexual behavior^48^.

The frequency of condom use is a key factor in the prevention of STIs, as it is widely recognized that unprotected sex increases the risk of acquiring these infections^2^. However, the results of this study diverge from the literature by suggesting that young people who use condoms more regularly are, paradoxically, more likely to test positive for syphilis. This phenomenon has precedents in international research, based on a US cohort that associated frequent condom use with syphilis test reactivity^25^. These findings require further investigation to clarify potential confounding factors, considering the scientific consensus on the effectiveness of condoms as a preventive method and the possible influence of information biases resulting from underreporting or omission by participants.

Based on the evidence presented, public policies aimed at preventing STIs and promoting sexual health among young people should prioritize multilevel interventions that combine health education, community strategies, and the use of digital technologies^49^ ^)-(^ ^50^. In this context, the Impact Triad^®^ experience demonstrates that peer-navigation-based approaches, which use trained community leaders to disseminate information and facilitate access to health services, significantly increase STI testing, condom use, and adherence to pre-exposure prophylaxis, in addition to strengthening social support and reducing discrimination among young people from minority groups^50^. 

At the same time, systematic reviews show that mobile health (mHealth) platforms, such as text messages, voice calls, and interactive apps, are well accepted by young people because they offer privacy, convenience, and adapted language. Therefore, it is additionally recommended that public policies integrate face-to-face and digital actions, promote the active participation of young people in the development of strategies, train health professionals and communities to address sensitive issues, and invest in continuous and intersectoral educational campaigns to enable universal and equitable access to sexual health prevention and care^49^.

In nursing and health practice, the findings call for priority screening for key groups among young people, educational interventions based on digital technologies, and the integration of psychosocial assessments into nursing protocols. Multidisciplinary action should combine TR (nursing and other health professionals duly trained for this purpose), coordination of protection networks (social assistance), and trauma management (psychology), with a focus on reducing inequalities in access to health and maximizing the promotion of sexual health.

Despite the limitations of this study, as the primary study focused exclusively on HIV, its analyses were limited to variables related to this infection. This methodological limitation resulted in the absence of specific data on VDRL, a crucial test for quantifying the serological burden of syphilis. The possibility of participant selection bias is assumed, since these are young people who sought care and are already familiar with Testing and Counseling Centers. Furthermore, convenience sampling in urban centers may underestimate the reality of young people in rural communities.

## Conclusion

The prevalence of syphilis-reactive RT in young people was estimated at 14.9%, a context that points to an intersectional structure permeated by multiple layers of vulnerabilities that associate the phenomenon with sociodemographic factors (male, transgender male or female, homosexual or pansexual), socio-structural (low family income, independent housing, and history of institutionalization), and behavioral (first non-consensual sexual intercourse, transactional sex, risky sexual practices, history of STIs, and drug use).

Furthermore, with regard to syphilis-reactive RT among young people, the logistic regression model showed that, while the 15-19 age group was a protective factor compared to the 20-24 age group, low income (less than two minimum wages) and male gender increased the chances of reactive RT. Notably, a previous history of STIs emerged as an important risk factor, increasing the chances of RT reactivity for the disease by 8.4 times.

Caution is advised when generalizing the results, particularly regarding the apparent ineffectiveness of condoms, which may reflect memory bias or underreporting of risky practices. Therefore, future studies should incorporate longitudinal designs and representative samples from different socioeconomic contexts.

## Data Availability

Datasets related to this article will be available upon request to the corresponding author.
