# Supplementary material for: Rapid syphilis testing among young people: prevalence and associated factors
Source: Rev Lat Am Enfermagem. 2026 Oct 7;34:e4958. doi: 10.1590/1518-8345.7488.4958 (PMC13679912; doi:10.1590/1518-8345.7488.4958)
Supplement: Translation

# Translation: Testagem rápida reagente para sífilis entre jovens: prevalência e fatores associados*

**DOI:** 10.1590/1518-8345.7488.4959

**Authors:** William Caracas Moreira, Leidyanny Barbosa de Medeiros, Rute Xavier Silva, Jordana de Almeida Nogueira, Mônica Oliveira Batista Oriá, Oriana Deyze Correia Paiva Leadebal

**Affiliations:** 1 Universidade Federal do Ceará, Campos Porangabuçu, Fortaleza, CE, Brasil.; 2 Universidade Federal da Paraíba, Campus I, João Pessoa, PB, Brasil.; 3 Bolsista da Coordenação de Aperfeiçoamento de Pessoal de Nível Superior (CAPES), Brasil.; 4 Bolsista do Conselho Nacional de Desenvolvimento Científico e Tecnológico (CNPq), Brasil.

**Keywords:** Sífilis, Adulto Jovem, Comportamento Sexual, Vulnerabilidade Sexual, Infecções Sexualmente Transmissíveis, Prevalência

## Abstract

**Objetivo:**

analisar a prevalência da testagem rápida reagente para sífilis entre jovens e seus fatores associados.

**Método:**

estudo transversal, desenvolvido em dois Centros de Testagem e Aconselhamento. Envolveu 301 jovens, cujas informações foram coletadas por instrumento validado e analisadas por estatística descritiva, testagens de qui-quadrado, razão de prevalência e regressão logística.

**Resultados:**

os jovens apresentaram prevalência de 14,9% para a testagem rápida reagente para sífilis, com associações significativas aos fatores sociodemográficos (sexo masculino, identidade trans e orientação sexual não heterossexual), socioestruturais (baixa renda, moradia independente e histórico de institucionalização) e comportamentais (sexo transacional, práticas sexuais de risco, histórico de infecções sexualmente transmissíveis e consumo de drogas). A análise multivariada confirmou maiores chances de testagem rápida reagente para sífilis entre jovens com baixa renda, sexo masculino e histórico de Infecções sexualmente transmissíveis, o que reforça a vulnerabilidade desses grupos.

**Conclusão:**

em suma, a prevalência e os fatores associados à testagem rápida reagente para sífilis entre jovens permeiam uma estrutura interseccional com múltiplas camadas de vulnerabilidades, contemplando dimensões sociodemográficas, socioestruturais e comportamentais.

## Destaques

**(1)** A prevalência da testagem rápida reagente para sífilis entre jovens foi estimada em 14,9%.

**(2)** Sexo masculino e IST prévias aumentam risco de testagem rápida reagente para sífilis.

**(3)** Fatores socioestruturais ampliam a vulnerabilidade à testagem rápida reagente para sífilis.

**(4)** Práticas sexuais de risco foram associadas à testagem rápida reagente para sífilis.

**(5)** Renda baixa e discriminação elevam chance de testagem rápida reagente para sífilis.

## Introdução

A sífilis é uma das Infecções Sexualmente Transmissíveis (IST) de notificação compulsória, causada pelo *Treponema pallidum* e transmitida por: via materno-fetal, durante a gestação; no momento do parto, por via sanguínea; ou através de relações sexuais sem a utilização adequada do preservativo^1^. Com diagnóstico acessível pelo Sistema Único de Saúde (SUS), a infecção pode ser rapidamente detectada e tratada para evitar evolução clínica, o surgimento de complicações, o aumento dos casos de sífilis congênita e de novos casos da infecção adquirida^2^. Ainda assim, estudos recentes destacam o caráter reemergente e um expressivo número de casos entre os jovens, em ascensão, especialmente, entre aqueles com menos de 24 anos^1^ ^)-(^ ^3^.

No que concerne às estratégias de diagnóstico precoce, tem-se a testagem rápida (TR) para sífilis^4^, a qual consiste em método imunocromatográfico capaz de detectar anticorpos contra o *Treponema pallidum* a partir de pequenas amostras de sangue, obtidas preferencialmente por punção digital, aspecto que promove agilidade e facilidade na coleta, inclusive em ambientes com pouca infraestrutura laboral^4^ ^)-(^ ^5^. O resultado, disponível em poucos minutos, pode ser classificado em reagente ou não reagente para sífilis. O resultado não reagente implica em reforço das medidas preventivas e possível repetição da TR em 30 dias, caso haja suspeita clínica ou risco recente. Por outro lado, resultados reagentes requerem a confirmação por teste não treponêmico, tal como o *Venereal Disease Research Laboratory* (VDRL), a fim de distinguir a infecção ativa da cicatriz sorológica^5^.

Compreendida pela adolescência e a vida adulta jovem, a juventude ocorre entre as idades de 15 a 24 anos, período em que as pessoas experienciam descobertas em diferentes aspectos, que por sua vez moldam relações sociais e contribuem para os comportamentos, dentre os quais, os sexuais^6^. Nessa perspectiva, estudos ressaltam que a expressão sexual das pessoas tem aflorado cada vez mais cedo e está comumente associada a déficits de conhecimento sobre IST e dificuldades dos pais e profissionais de saúde em abordar a temática na população, conforme as condições de vulnerabilidade para a sífilis entre jovens^6^ ^)-(^ ^7^.

Mundialmente, o estudo *Global Burden of Disease* demonstrou que, embora a sífilis não figure como a IST mais incidente em números absolutos, sua taxa padronizada por idade apresentou crescimento contínuo entre 1990 e 2021, dentre os quais destaca-se o Brasil, que registrou o maior aumento dessa taxa entre os países analisados^8^.

Nesse contexto, detalha-se que, entre 2011 e 2022, o Brasil notificou mais de 1,1 milhão de casos de sífilis adquirida, dos quais 36% ocorreram em jovens. Entre 2020 e 2021, a taxa de detecção nacional subiu de 59,1 para 78,5 casos por 100 mil habitantes, tendência observada também na região Nordeste, cujos índices saltaram de 29,9 para 47,3 casos por 100 mil habitantes. A Paraíba, estado nordestino, demonstrou crescimento semelhante ao padrão regional, ainda que sem ultrapassar a média nacional^2^. Além disso, cabe ressaltar que os dados de notificações compulsórias para sífilis, na Paraíba, seguem a tendência global^9^, isto é, diminuíram desde o início da pandemia da COVID-19, o que certamente não reflete redução da ocorrência da infecção, mas fragilidade no diagnóstico, subnotificação e, consequentemente, ausência do tratamento oportuno^10^.

Este estudo, além de valorizar a TR para sífilis no contexto da saúde pública^5^, põe em evidência a vulnerabilidade de faixa etária específica (jovens de 15 a 24 anos) à sífilis, diferenciando-se dos demais estudos que, majoritariamente, abordam populações gerais, gestantes ou grupos-chave ligados, sobretudo, à identidade de gênero e/ou orientação sexual, conforme revisão sistemática da literatura^11^. 

Aliadas à necessidade científica para contribuir com as metas dos Objetivos de Desenvolvimento Sustentável (ODS), da Agenda 2030 - Organização das Nações Unidas (ONU)^12^, a produção de estudos de prevalência da sífilis adquirida entre jovens encontra sólida justificativa tanto no cenário epidemiológico global e brasileiro^2^ ^),(^ ^8^ quanto na dinâmica da pesquisa em saúde^7^. 

Diante do exposto, o objetivo deste estudo foi analisar a prevalência da TR reagente para sífilis entre jovens e seus fatores associados.

## Método

### Tipo ou delineamento do estudo

Estudo transversal, desenvolvido a partir de recorte da pesquisa primária intitulada “HIV na população jovem: subsídios para o enfrentamento da epidemia a partir da análise de fatores socioestruturais e comportamentais”, reportado segundo as recomendações do *Strengthening the Reporting of OBservational studies in Epidemiology* (STROBE)^13^.

### Local do estudo

Desenvolvido em dois Centros de Testagem e Aconselhamento de dois municípios-sede das macrorregiões de saúde I e II, do estado da Paraíba, Brasil. Esses serviços funcionam por demanda espontânea ou referenciamento de outros serviços, nos turnos matutino e vespertino, atendem 134 dos 223 municípios do estado e ofertam ações de promoção, prevenção, diagnóstico e tratamento em saúde sexual. 

### Período

A coleta de dados ocorreu de fevereiro a julho de 2021.

### População

A população foi composta por jovens com idades entre 15 e 24 anos. 

### Definição da amostra

A amostra foi definida por cálculo para populações finitas, considerou-se estimativa média mensal de TR realizada nos serviços investigados (Sede da Macrorregião I- N=500; Sede da Macrorregião II- N=320); nível de significância de 5%; índice de confiança (IC) de 95%; e um valor antecipado de Prevalência (P) baseado na taxa de detecção de casos de HIV/aids na faixa etária preconizada, com foco na pesquisa original (Sede da Macrorregião I com P = 23,0%; Sede da Macrorregião I com P = 8,6%); o que definiu a amostra em 301 jovens, dos quais 200 são Sede da Macrorregião I e 101 Sede da Macrorregião II.

### Critérios de seleção

A seleção dos participantes ocorreu por amostragem não probabilística, adotou-se o critério de conveniência em função das particularidades do local de estudo. Foram incluídas as pessoas cujo *status* sorológico para sífilis era desconhecido no momento da pesquisa primária e que buscaram os Centros de Testagem e Aconselhamento a fim de realizar a TR para alguma das IST. Ademais, foram excluídos aqueles que apresentavam alguma deficiência que os impossibilitasse de responder ao formulário.

### Instrumento utilizado e variáveis do estudo

O instrumento foi construído e validado por pesquisadores do Núcleo de Estudos em HIV, Saúde e Sexualidade (NEHAS)^14^. Estruturado em 45 questões distribuídas em quatro segmentos: 1 - Identificação da entrevista; 2 - Variáveis sociodemográficas (idade, sexo, aspectos inerentes à sexualidade, cor autodeclarada, anos de estudo); 3 - Variáveis socioestruturais (trabalho, *status* de relacionamento atual, condições de moradia, renda familiar - considerou-se o salário-mínimo de R$ 1.100,00 (vigente em 2021), histórico de institucionalização, discriminação, tratamento psiquiátrico e vitimização por violência sexual); e 4 - Variáveis comportamentais (coitarca, histórico de IST, consentimento em relações sexuais, aspectos relacionados às práticas sexuais, sexo transacional, consumo de drogas)^14^.

### Coleta de dados

A coleta de dados ocorreu nos turnos da manhã e tarde, de acordo com a disponibilidade dos pesquisadores, variando conforme os dias da semana, até a obtenção da amostra pretendida. 

Conforme estabelecido nos critérios de inclusão e exclusão, os jovens que buscaram os Centros de Testagem e Aconselhamento foram informados sobre o estudo e convidados a participar. Para o aceite foi necessária a assinatura do Termo de Consentimento Livre Esclarecido ou o Termo de Assentimento Livre Esclarecido para menores de 18 anos. Por conseguinte, participaram de entrevista individualizada, em ambiente privativo, orientada pelo instrumento de coleta de dados^14^ com abordagem face-a-face por pesquisador treinado para conduzi-la. Ressalta-se que esse processo antecedeu a TR do(a) participante.

### Tratamento e análise dos dados

Os dados de interesse deste estudo foram transportados do banco da pesquisa original e organizados em planilha do Microsoft Excel^®^, para compilação e recategorização por meio do programa estatístico *Statistical Package for the Social Sciences* (SPSS), versão 20.0. 

Para a análise da caracterização dos participantes, calculou-se as frequências absolutas e relativas, e medidas de tendência central e de dispersão. 

Para análise da prevalência, considerou-se o número de TR reagentes para sífilis, dividido pelo total amostral (n= 301). Para cálculo de qui-quadrado (X²) de Pearson e razão de prevalência (RP) considerou-se a associação do desfecho TR reagente para sífilis e os fatores associados. Ressalta-se que para essas análises, excluíram-se os dados referentes às categorias: “ignorado”; “não quis responder”; e “não soube responder”.

Para a análise multivariada, optou-se por utilizar o modelo de regressão logística, adotando-se o *backward* a fim de selecionar as variáveis que melhor explicassem o fenômeno em estudo, considerando o nível de significância de 5% (p < 0,05)^15^.

### Aspectos éticos

Este estudo seguiu os preceitos éticos da pesquisa que envolve seres humanos previstos na Resolução n° 466/12 do Conselho Nacional de Saúde; foi aprovado pelo Comitê de Ética em Pesquisa do Centro de Ciências da Saúde da Universidade Federal da Paraíba - Parecer nº 3.935.713 (26/03/2020), tanto quanto se conduziu os procedimentos necessários à garantia da proteção das informações acessíveis à equipe de pesquisa e do anonimato por ocasião da publicação dos resultados.

## Resultados

Dos 382 jovens abordados pelos pesquisadores durante a coleta de dados, 301 aceitaram participar. Os participantes possuíam idade média de 20,83 anos (desvio-padrão ± 2,3), cuja caracterização sociodemográfica indicou predominância da faixa etária de 20 a 24 anos (212/70,4%), sexo masculino (155/51,5%); cor autodeclarada não-branca (217/72,1%); pessoas cisgênero (291/96,6%), heterossexuais (175/58,1%), com mais de oito anos de estudo (259/86%), e quanto às que atualmente não estudam, encontrou-se um percentual de 86% (163). 

Aos fatores intrínsecos à dimensão socioestrutural, houve predomínio de jovens com relacionamento fixo (172/57,3%), renda familiar entre um e dois salários mínimos (111/37,2%), que não trabalham (166/55,1%) e dividem moradia apenas com familiares (152/50,5%). Também relataram vitimização decorrente de discriminação por orientação sexual (104/34,7%) e por identidade de gênero (57/18,9%), principalmente em escolas e universidades (86/82,7% e 11/19,6%, respectivamente). Além disso, foi reportada violência sexual (84/27,9%) - perpetrada principalmente por desconhecidos (29/35%), histórico de tratamento psiquiátrico (61/20,3%) e prisional ou de institucionalização (10/3,3%). 

No perfil comportamental dos jovens, observou-se o predomínio das seguintes características: coitarca até os 15 anos (153/50,8%) - prática não consentida por uma parcela dos jovens (15/5%); utilizar o preservativo numa frequência de “às vezes” (167/55,4%); praticar sexo oral (237/78,7%) e vaginal (214/71,1%); e uso de bebidas alcoólicas (174/57,8%), destes, uma parcela referiu (68/39,5%) já ter deixado de utilizar o preservativo quando sob efeito de bebidas alcoólicas; outra, afirmou que não lembra se fez ou não uso do preservativo nesse tipo de ocasião (11/6,4%).

Houve relatos de sexo transacional (29/9,6%), prática sexual anal insertiva (114/37,9%) e receptiva (124/41,2%), diagnóstico prévio de IST (63/20,9%), dificuldade em adquirir (32/10,6%) ou negociar o preservativo (103/34,2%) e consumo de drogas ilícitas também foi reportado (103/34,2%).

Quanto à prevalência e associações inerentes aos resultados, identificou-se prevalência de TR reagente para sífilis em parcela de jovens (45/14,9%) e associação com fatores sociodemográficos, socioestruturais e comportamentais.

No tocante aos fatores sociodemográficos, o desfecho para a TR reagente para sífilis foi associado às variáveis pertinentes ao sexo, identidade de gênero e orientação sexual (Tabela 1). Nessa dimensão (Tabela 1), o sexo masculino apresentou RP de 3,8 (IC95%: 1,9-7,5) vezes maior para TR reagente para sífilis, assim como os jovens cuja a identidade de gênero era homem trans ou mulher trans tiveram, respectivamente, RP de 8,0 (IC95%: 1,7-36,9) e 10,1 (IC95%: 4,4-23,1). Ademais, ser homossexual ou pansexual mostrou, respectivamente, RP de 4,4 (IC95%: 2,3-8,4) e 4,8 (IC95%: 1,6-14,2), o que indica maior vulnerabilidade à TR reagente para sífilis nesses grupos.

**Tabela 1:** Prevalência (P*), razão de prevalência (RP^†^) e testagem de qui-quadrado de associação entre o desfecho da testagem rápida reagente para sífilis e fatores sociodemográficos dos jovens (n = 301). João Pessoa, PB, Brasil, 2021

Variáveis		Testagem rápida reagente para sífilis (N)		P* (%)	RP^†^ [IC^‡^ 95,0%]	**X² *p*-valor** ^§^
		Sim	Não			
Faixa etária	15-19 anos	08	81	9,0	1	0,060
	20-24 anos	37	175	17,5	1,9 [0,9; 4,0]	
Sexo	Feminino	09	137	6,2	1	0,001^||^
	Masculino	36	119	23,2	3,8 [1,9; 7,5]	
Cor autodeclarada	Branco	12	72	14,3	1	0,841
	Não branco	33	184	15,2	1,00 [0,6; 1,9]	
Anos de estudo	≤ 8 anos	04	38	9,5	1	0,288
	> 8 anos	41	218	15,8	1,7 [0,6; 4,4]	
Estuda	Sim	19	119	13,8	1	0,597
	Não	26	137	16,0	1,2 [0,6; 2,0]	
Identidade de gênero	Homem	30	116	20,5	3,3 [1,6; 6,7]	0,001^||^
	Mulher	09	136	6,2	1	
	Homem trans	01	1	50,0	8,0 [1,7; 36,9]	
	Mulher trans	05	3	62,5	10,1 [4,4; 23,1]	
Orientação sexual	Heterossexual	12	163	6,9	1	0,001^||^
	Homossexual	24	55	30,4	4,4 [2,3; 8,4]	
	Bissexual	06	32	15,8	2,0 [0,9; 5,7]	
	Panssexual	03	6	33,3	4,8 [1,6; 14,2]	

*P = Prevalência; ^†^RP = Razão de prevalência; ^‡^IC = Intervalo de Confiança; ^§^ *X*² *p* valor = *p* valor do teste Qui-quadrado para a comparação de proporção; ^||^p-valor <0,05 = Associação entre a testagem rápida reagente para sífilis e a variável correspondente

Na dimensão compreendida por fatores socioestruturais, verificou-se a associação estatística entre a TR reagente para sífilis e as seguintes variáveis: renda familiar, com quem divide moradia, vitimização decorrente de discriminação por identidade de gênero e orientação sexual, e já ter sido preso(a) ou institucionalizado(a). Assim, salienta-se que a renda familiar inferior a um salário mínimo elevou as RP para 2,3 (IC95%: 1,2-4,6) vezes para TR reagente para sífilis. Além disso, a moradia apenas com amigos ou sozinho apresentaram, respectivamente, RP 3,9 (IC95%: 1,9-8,0) e 3,2 (IC95%: 1,6-6,3). Fenômeno semelhante ocorre com jovens que possuíam histórico de prisão/institucionalização com RP de 2,8 (IC95%: 1,7-6,4) (Tabela 2).

**Tabela 2:** Prevalência (P*), razão de prevalência (RP^†^) e testagem de qui-quadrado de associação entre o desfecho da testagem rápida reagente para sífilis e fatores socioestruturais dos jovens (n = 301). João Pessoa, PB, Brasil, 2021

Variáveis		Testagem rápida reagente para sífilis (N)		P* (%)	RP^†^ [IC^‡^ 95,0%]	**X² *p* valor** ^§^
		Sim	Não			
Renda familiar	≤ 1 SM^||^	23	70	24,7	2,3 [1,2; 4,6]	0,008^¶^
	1 a 2 SM^||^	12	99	10,8	1,0 [0,5; 2,2]	
	> 2 SM^||^	10	84	10,6	1	
Trabalha	Sim	20	115	14,8	1	0,953
	Não	25	141	15,1	1,0 [0,6; 1,7]	
Com quem divide moradia	Apenas com amigos	08	13	38,1	3,9 [1,9; 8,0]	0,001^¶^
	Sozinho (a)	12	26	31,6	3,2 [1,6; 6,3]	
	Apenas com parceiro (a)	07	37	15,9	1,6 [0,7; 3,7]	
	Apenas com familiares	15	137	9,9	1	
	Parceiro (a) e amigos ou familiares	03	43	6,5	0,7 [0,2; 2,2]	
*Status* de relacionamento	Fixo	17	155	9,9	1	0,051
	Casual	11	33	25,0	2,5 [1,3; 5,0]	
	Com mais de uma parceria	02	12	14,3	1,4 [0,3; 5,6]	
	Sem relacionamento	15	55	21,4	2,1 [1,1; 4,1]	
Discriminação por identidade de gênero	Sim	15	42	26,3	2,1 [1,2; 3,7]	0,008^¶^
	Não	30	214	12,3	1	
Discriminação por orientação sexual	Sim	29	75	27,9	3,4 [1,9; 6,0]	0,001^¶^
	Não	16	179	8,2	1	
Vitimização por violência sexual	Sim	16	68	19,0	1,4 [0,8; 2,5]	0,215
	Não	29	188	13,4	1	
Já foi preso ou institucionalizado	Sim	04	6	40,0	2,8 [1,7; 6,4]	0,024^¶^
	Não	41	250	14,1	1	
Histórico de tratamento psiquiátrico	Sim	11	50	18,0	1,3 [0,7; 2,3]	0,450
	Não	34	206	14,2	1	

*P = Prevalência; ^†^RP = Razão de prevalência; ^‡^IC = Intervalo de Confiança; ^§^ *X*² *p* valor = *p* valor do teste Qui-quadrado para a comparação de proporção; ^||^SM = Salário-mínimo de R$ 1.100,00, Brasil, vigente em 2021; ^¶^p-valor <0,05 = Associação entre a testagem rápida reagente para sífilis e a variável correspondente

Concernente aos fatores comportamentais, a TR reagente para sífilis foi associada ao não consentimento na primeira relação sexual, frequência do uso do preservativo, sexo transacional, práticas de sexo vaginal, anal insertivo e receptivo, histórico de IST e consumo de drogas ilícitas (Tabela 3). Logo, observa-se que o não consentimento na primeira relação sexual aumentou a RP em 2,3 (IC95%: 1,1-5,1) para TR reagente para sífilis, assim como as práticas sexuais: sexo transacional RP em 3,8 (IC95%: 2,2-6,4), sexo anal insertivo RP em 2,6 (IC95%: 1,5-4,6) e receptivo RP em 1,7 (IC95%: 1,1-3,0). Além disso, os jovens que não realizavam sexo vaginal apresentaram RP 3,8 (IC95%: 2,2-6,6) vezes maior para TR reagente para sífilis (Tabela 3).

**Tabela 3:** Prevalência (P*), razão de prevalência (RP^†^) e testagem de qui-quadrado de associação entre o desfecho da testagem rápida reagente para sífilis e fatores comportamentais dos jovens (n = 301). João Pessoa, PB, Brasil, 2021

Variáveis		Testagem rápida reagente para sífilis (N)		P* (%)	RP^†^ [IC^‡^ 95,0%]	**X² *p*-valor** ^§^
		Sim	Não			
Coitarca (n= 294)	≤ 15 anos	25	128	16,3	1,1 [0,6; 2,0]	0,608
	> 15 anos	20	121	14,2	1	
Primeira relação sexual consentida (n= 298)	Sim	40	243	14,1	1	0,043^||^
	Não	05	10	33,3	2,3 [1,1; 5,1]	
Dificuldade de adquirir preservativos (n= 299)	Sim	08	24	25,0	1,8 [0,9; 3,5]	0,096
	Não	37	230	13,9	1	
Frequência de uso do preservativo (n= 297)	Sempre	21	40	34,4	5,9 [2,1; 16,3]	0,001^||^
	Às vezes	20	147	12,0	2,0 [0,7; 5,8]	
	Nunca	04	65	5,8	1	
Dificuldade de negociação do preservativo (n= 294)	Sempre	03	28	9,7	1	0,265
	Às vezes	15	57	20,8	2,1 [0,6; 6,9]	
	Nunca	27	164	14,1	1,4 [0,4; 4,5]	
Sexo transacional	Sim	13	16	44,8	3,8 [2,2; 6,4]	0,001^||^
	Não	32	240	11,8	1	
Pratica sexo vaginal (n=297)	Sim	18	196	8,4	1	0,001^||^
	Não	27	56	32,5	3,8 [2,2; 6,6]	
Prática sexo oral (n=297)	Sim	38	199	16	1,3 [0,6; 2,9]	0,399
	Não	7	53	11,7	1	
Prática sexo anal insertivo (n=297)	Sim	28	86	24,6	2,6 [1,5; 4,6]	0,001^||^
	Não	17	166	9,3	1	
Prática sexo anal receptivo (n=297)	Sim	25	99	20,2	1,7 [1,1; 3,0]	0,041^||^
	Não	20	153	11,6	1	
Histórico de IST^¶^ (n= 289)	Sim	30	33	47,6	7,1 [4,1; 12,5]	0,001^||^
	Não	15	211	6,6	1	
Consome bebida alcoólica	Sim	31	143	17,8	1,6 [0,9; 2,9]	0,103
	Não	14	113	11,0	1	
Consome drogas ilícitas	Sim	23	80	22,3	2,0 [1,2; 3,4]	0,010^||^
	Não	22	176	11,1	1	

*P = Prevalência; ^†^RP = Razão de prevalência; ^‡^IC = Intervalo de Confiança; ^§^ *X*² *p* valor = *p* valor do teste qui-quadrado para a comparação de proporção; ^||^p-valor <0,05 = Associação entre a testagem rápida reagente para sífilis e a variável correspondente; ^¶^IST = Infecções Sexualmente Transmissíveis

Ainda nesse contexto, o histórico prévio de IST e o consumo de drogas ilícitas aumentaram a RP em, respectivamente, 7,1 (IC95%: 4,1-12,5) e 2,0 (IC95%: 1,2-3,4) para TR reagente para sífilis. Quanto à frequência de uso do preservativo, aqueles que responderam sempre utilizar apresentaram RP 5,9 (IC 2,1-16,3) vezes maior para a TR reagente para sífilis em comparação com os jovens que responderam utilizar às vezes ou nunca (Tabela 3).

Com base na análise por regressão logística, ser jovem com faixa etária de 15 a 19 anos constitui efeito protetor da TR reagente para sífilis quando comparada a jovens de 20 a 24 anos. Entretanto, aqueles com renda inferior a um salário mínimo ou entre um e dois salários mínimos apresentaram 2,9 e 2,4, respectivamente, mais chances para TR reagente para sífilis, comparados aos jovens de renda superior a três salários mínimos. De igual maneira, ser do sexo masculino aumentou três vezes as chances para TR reagente para sífilis, comparado ao sexo feminino, e possuir histórico de alguma IST representou 8,4 mais chances de TR reagente para sífilis, em relação aos que não relataram tal histórico (Tabela 4).

**Tabela 4:** Modelo de regressão logística contemplando fatores associados à testagem rápida reagente para sífilis entre jovens (n = 301). João Pessoa, PB, Brasil, 2021

Variáveis	Coeficiente	Erro-padrão	*Odds ratio*	Intervalo de confiança (95%)	*p* valor*
Faixa etária de 15-19 anos	-0,78	0,34	0,45	[0,23 - 0,89]	0,02^†^
Sexo masculino	1,11	0,43	3,06	[1,31 - 7,11]	0,01^†^
Renda familiar ≥ 3 SM^‡^					0,01^†^
Renda familiar ≤ 1 SM^‡^	1,09	0,42	2,98	[1,31 - 6,80]	0,01^†^
Renda familiar entre 1-2 SM^‡^	0,90	0,46	2,47	[1,01 - 6,09]	0,04^†^
Ter diagnóstico de IST^§^ anterior	2,13	0,36	8,46	[4,11 - 17,42]	0,01^†^
**R² (Nagelkerke)** ^||^					70,5%
**Teste de Hosmer-Lemeshow (p-valor)**					0,20

* *p* -valor = *p* valor do Modelo de regressão logística; ^†^p<0,05 = Associação entre a testagem rápida reagente para sífilis e os fatores investigados; ^‡^SM = Salário-mínimo vigente = R$ 1.100,00, Brasil, 2021; ^§^IST = Infecção Sexualmente Transmissível; ^||^R² (Nagelkerke) = Mede o desempenho do modelo a partir das variáveis utilizadas

## Discussão

O perfil sociodemográfico, apresentado neste estudo, assemelha-se à literatura nacional^16^ ^)-(^ ^17^ e internacional^7^ ^),(^ ^18^ ^)-(^ ^23^ relacionada à prevalência de jovens do sexo masculino^16^ ^)-(^ ^19^ ^),(^ ^21^ ^)-(^ ^22^, cisgênero^7^, heterossexuais^7^ ^),(^ ^16^, faixa etária de 20 a 24 anos^20^, não brancos^16^ ^),(^ ^19^ ^),(^ ^23^, e com mais de oito anos de estudo, em suas amostras^16^. Destoou-se apenas a variável “estuda atualmente”, pois a maioria das pesquisas que focalizam jovens tendem a abordar o cenário universitário, o que consequentemente influenciou a variável em questão^24^.

Os jovens do sexo masculino assumem o maior risco não só para a TR reagente para sífilis como a outras IST^21^ ^)-(^ ^22^, isto posto, explicita-se que a vulnerabilidade pode estar ligada à comportamentos como a busca por parcerias sexuais por meio da internet, idade precoce na coitarca, maior frequência de relações sexuais, baixa adoção de práticas de autocuidado e preservação de saúde sexual, quando comparados ao sexo feminino^17^ ^)-(^ ^18^ ^),(^ ^21^ ^)-(^ ^22^.

Jovens de 20 a 24 anos apresentam maior vulnerabilidade à TR reagente para sífilis e outras IST, especialmente as do sexo feminino, devido à convergência de fatores sociodemográficos e econômicos desfavoráveis, práticas sexuais transacionais e uso inconsistente de preservativos^25^ ^)-(^ ^26^. Nesse cenário, a persistência da desinformação sobre saúde sexual e a exposição a comportamentos de risco amplificam essa vulnerabilidade, fundamental para o acesso a informações adequadas e a análise coletiva dos fatores que interferem nesse processo^27^.

Para além dos fatores individuais, as análises bi e multivariadas, apresentadas neste estudo, sugerem a vulnerabilidade de grupos populacionais ligados ao meio LGBTQIAPN+, uma vez que apontam o maior risco para TR reagente para sífilis em pessoas do sexo masculino, transsexuais, homossexuais ou pansexuais. Essa conjuntura sociodemográfica pode estar ligada à desinformação acarretada por uma cascata de eventos embasadas no preconceito estrutural e histórico, vivenciado por esse grupo, cujos impactos são percebidos no acesso aos serviços de saúde, à disponibilidade de recursos e informações para prevenir comportamentos sexuais de risco, IST, e promover a saúde sexual^28^ ^)-(^ ^30^.

Ainda, as variáveis socioestruturais refletem padrões complexos de vulnerabilidade nesse grupo populacional, com destaque para interseccionalidades entre condições econômicas, situações de violência e saúde mental^31^. Essas condições encontram-se consolidadas na literatura quando visualizadas de forma distinta^32^, contudo, a evidência da conjuntura, com suas interseccionalidades, em estudos de prevalência ligados ao risco de TR reagente para sífilis ainda é pouco explorado.

Nesse contexto, apesar do potencial de transformação social dos ambientes educacionais em questões de respeito e dignidade humana, escolas e universidades perpetuam uma estrutura preconceituosa, o que configura espaços de reprodução de violências LGBTfóbicas^29^ ^),(^ ^33^. Outro fator intrínseco à vulnerabilidade socioestrutural desses jovens está ligada ao histórico de privação de liberdade, particularmente relevante em populações jovens periféricas que afetam a forma como os jovens se relacionam (ao assumir comportamentos sexuais de risco), inserção no mercado de trabalho e como percebem o acesso à saúde^34^.

Outrossim, o relato de violência sexual de adolescentes e jovens está presente desde grandes centros urbanos a comunidades rurais^35^, cuja predominância de perpetradores desconhecidos, apontada neste estudo, contrasta parcialmente com estudo, realizado no interior do Piauí, que apontou parceiros íntimos e amigos como os principais perpetradores, o que sugere ser a realidade de contextos específicos de violência urbana ou subnotificação de casos intrafamiliares^36^.

A renda familiar é um fator socioestrutural inerente ao acesso à saúde e informação, visto que quão maior a renda, melhor o acesso aos serviços e ações de saúde^37^. Isto posto, estudos demonstram que, quanto mais baixa a renda familiar, maior a probabilidade do uso inconsistente de preservativos e, consequentemente, maior o risco para IST^31^ ^),(^ ^37^ ^)-(^ ^38^.

Quanto às variáveis “não trabalha atualmente” e “não estuda atualmente”, a literatura elucida que os jovens vivenciam aspectos típicos da adultez emergente, permeada por marcadores sociais como gênero, escolaridade e experiência de trabalho, além de serem afetados pela precariedade do mercado laboral^23^. Essa condição, além de limitar oportunidades de desenvolvimento pessoal e social, expõe esses jovens a múltiplas situações de vulnerabilidade. Tais fatores estão associados a contextos de maior isolamento social, dificuldade de acesso a informações e serviços de saúde, e à adoção de comportamentos de risco, o que torna essa população jovem especialmente suscetível às IST^23^.

Em nossa amostra, pelo menos metade dos jovens relataram morar apenas com familiares, o que com base na literatura constitui fator de proteção às IST^39^ ^)-(^ ^40^. Entretanto, aqueles cuja moradia se dá com amigos e/ou sozinho representou maior risco para TR reagente para sífilis e para outras IST, visto que o jovem possui maior liberdade e pouca supervisão, ao assumir, portanto, comportamentos sexuais de risco^39^. Ademais, salienta-se que a faixa etária (15-19 anos) entendida como fator de proteção à TR reagente para sífilis, neste estudo, pode ser explicada pela moradia com familiares, alta supervisão e coitarca tardia^40^.

Em relação aos fatores comportamentais, observa-se que o contexto relacional dos jovens determina padrões comportamentais que influenciam diretamente as dinâmicas de proteção sexual e cria cenários de vulnerabilidade epidemiológica diferenciados^24^. Contudo, o risco estimado pelo *status* de relacionamento assumido por jovens às IST ainda não é consenso científico. Isto posto, análises comportamentais demonstram que parceiros casuais apresentam maior propensão à negligência profilática - seja por esquecimento ou despreocupação deliberada - enquanto relacionamentos fixos favorecem a substituição progressiva do preservativo por critérios de confiança interpessoal^21^ ^),(^ ^24^ ^),(^ ^41^. Nesse sentido, ambos os modelos podem elevar a exposição à sífilis e outras IST, pois a redução no uso consistente de barreiras físicas facilita a transmissão durante práticas sexuais, independentemente da percepção subjetiva de segurança^24^.

Uma revisão de escopo mostrou que a iniciação sexual entre jovens ocorre, em média, aos 15 anos^24^. Nesse contexto, a primeira experiência sexual assume relevância epidemiológica, pois a ausência de preservativo e/ou a falta de consentimento potencializam comportamentos de risco e aumentam a vulnerabilidade às IST^21^ ^),(^ ^24^. Assim, a coitarca desprotegida ou não consensual configura um importante fator para a exposição e perpetuação dessas infecções entre os jovens^21^ ^),(^ ^42^. 

No que diz respeito à dimensão do conhecimento anteriormente mencionado, percebe-se que a coerção e o abuso sexual não estão relacionados apenas a gravidez indesejada, mas também ao risco às IST. Por sua vez, a incidência de relações sexuais não consensuais é um indicador das desigualdades de gênero e dos padrões de masculinidade predominante^31^. Assim, vê-se que a iniciação sexual coagida e forçada está associada ao uso inconsistente de preservativos, experiências recentes de violência e resultados de saúde mental entre jovens, conjuntura que explicita as múltiplas camadas de vulnerabilidade dos jovens, especialmente as do sexo feminino^31^ ^),(^ ^42^. 

Reconhece-se, na atualidade, que a prática sexual transacional se traduz em fator de vulnerabilidade comportamental, de interfaces sociais, econômicas e/ou estruturais^21^ ^),(^ ^24^. Nesse sentido, um estudo apontou que jovens em situação de rua frequentemente negligenciam o uso de preservativos durante relações sexuais transacionais devido a pressões de parceiros íntimos, necessidades financeiras imediatas e medo de violência, isso descreve cenário propício para a transmissão da sífilis e outras IST^43^. 

A dinâmica, anteriormente mencionada, reproduz-se em diferentes contextos: entre universitários sul-africanos, 1,4% dos 918 pesquisados relataram engajar-se nessa prática^44^; enquanto em adolescentes norte-americanos do ensino médio, a prevalência atingiu 7,4%, com destaque para o sexo masculino (2,5 vezes mais propenso)^45^. Adicionalmente, fatores estruturais como pobreza, exclusão social, acesso limitado a serviços de saúde perpetuam ciclos de vulnerabilidade e dificulta tanto a prevenção quanto o diagnóstico precoce dessas infecções^24^ ^),(^ ^43^.

Entre as práticas sexuais assumidas pelos jovens, o sexo anal emerge como fator de maior predisposição à TR reagente para sífilis, outras IST^24^, pela combinação de aspectos como: maior fragilidade do epitélio retal a infecções; menor percepção de risco, cujo sentimento de invulnerabilidade viabiliza a adoção de comportamentos sexuais de risco; e déficits no acesso às informações adequadas e a serviços de saúde^46^.

Está consolidado na literatura que o histórico prévio de IST indica exposição anterior a comportamentos de risco, os quais tendem a se repetir ao longo do tempo^21^ ^),(^ ^24^. Revisões da literatura demonstram associação significativa entre antecedentes de IST e maior risco para TR reagente para sífilis e outras infecções, corroborando com a persistência de vulnerabilidades comportamentais^21^ ^),(^ ^24^. Nesse sentido, detalha-se o fenômeno por estudos que evidenciaram que pessoas com história de IST apresentam risco 1,8 vezes maior de adquirir outras IST^27^, enquanto o diagnóstico prévio de duas outras IST eleva em 5,83 vezes a probabilidade de infecção por sífilis^47^.

Noutro contexto, a associação entre o consumo de álcool e/ou de drogas ilícitas e práticas sexuais inseguras entre jovens configura um eixo crítico para a transmissão de sífilis e outras IST. Um estudo apontou que o uso de substâncias psicoativas altera padrões de julgamento, reduz a percepção de risco durante interações sociais - em eventos festivos os jovens encontram-se menos preocupados com os riscos subsequentes e, consequentemente, assumem comportamentos sexuais de risco^48^.

A frequência de uso do preservativo representa fator central na prevenção de IST, visto que é amplamente reconhecido que a prática sexual desprotegida eleva o risco de aquisição dessas infecções^2^. No entanto, os resultados deste estudo divergem da literatura ao sugerir que jovens que utilizam preservativo com maior regularidade, contraditoriamente, apresentam maior probabilidade de TR reagente para sífilis. Tal fenômeno encontra precedente em pesquisa internacional, a partir de uma coorte norte-americana que associou o uso frequente de preservativos com a reatividade de testes para sífilis^25^. Esses achados demandam investigações adicionais para esclarecer potenciais fatores de confusão, deve considerar o consenso científico sobre a eficácia do preservativo como método preventivo e a possível influência de vieses de informação decorrentes de subnotificação ou omissão por parte dos participantes.

Com base nas evidências apresentadas, políticas públicas voltadas à prevenção de IST e promoção da saúde sexual entre jovens devem priorizar intervenções multiníveis que combinem educação em saúde, estratégias comunitárias e o uso de tecnologias digitais^49^ ^)-(^ ^50^. Nesse contexto, a experiência do *Impact Triad* ^*®*^ demonstra que abordagens baseadas em navegação de pares, que utilizam líderes comunitários treinados para disseminar informações e facilitar o acesso a serviços de saúde, aumentam significativamente a realização de TR para IST, o uso de preservativos e a adesão à profilaxia pré-exposição, além de fortalecer o apoio social e reduzir a discriminação entre jovens de grupos minoritários^50^. 

Paralelamente, revisão sistemática evidencia que plataformas de saúde móvel (*mHealth*), como mensagens de texto, chamadas de voz e aplicativos interativos, são bem aceitas pelos jovens por oferecerem privacidade, conveniência e linguagem adaptada. Portanto, adicionalmente, recomenda-se que políticas públicas integrem ações presenciais e digitais, promovam a participação ativa de jovens na elaboração das estratégias, capacitem profissionais de saúde e comunidades para abordar temas sensíveis, e invistam em campanhas educativas contínuas e intersetoriais, para possibilitar acesso universal e equitativo à prevenção e ao cuidado em saúde sexual^49^.

Na prática de enfermagem e saúde, os achados demandam triagem prioritária para grupos-chave entre os jovens, intervenções educativas baseadas em tecnologias digitais e integração de avaliações psicossociais nos protocolos de enfermagem. A atuação multidisciplinar deve combinar TR (enfermagem e demais profissionais de saúde devidamente capacitados para tal), articulação de redes de proteção (assistência social) e manejo de traumas (psicologia), com foco em reduzir iniquidades no acesso à saúde e maximizar a promoção da saúde sexual.

A despeito das limitações deste estudo, como o estudo primário focou exclusivamente no HIV, suas análises limitaram-se às variáveis relacionadas a essa infecção. Essa delimitação metodológica resultou na ausência de dados específicos do VDRL, exame crucial para quantificação da carga sorológica da sífilis. Assume-se a possibilidade de viés de seleção dos participantes, visto que se trata de jovens que buscaram atendimento e já conhecem os Centros de Testagem e Aconselhamento. Ademais, a amostragem por conveniência em centros urbanos pode subestimar a realidade de jovens de comunidades rurais.

## Conclusão

A prevalência de TR reagente para sífilis em jovens foi estimada em 14,9%, cujo contexto aponta uma estrutura interseccional permeada por múltiplas camadas de vulnerabilidades que associa o fenômeno aos fatores sociodemográficos (sexo masculino, ser homem ou mulher trans e homossexual ou pansexual), socioestruturais (baixa renda familiar, moradia independente e histórico de institucionalização) e comportamentais (primeira relação sexual não consentida, sexo transacional, práticas sexuais de risco, histórico de IST e consumo de drogas).

Ainda, no que tange à TR reagente para sífilis entre jovens, o modelo de regressão logística mostrou que, enquanto a faixa etária de 15 a 19 anos configurou-se como fator protetor em comparação ao grupo de 20 a 24 anos, a baixa renda (inferior a dois salários mínimos) e o sexo masculino aumentaram as chances para a TR reagente. Notadamente, o histórico prévio de IST despontou-se como importante fator de risco, elevando 8,4 vezes as chances de TR reagente para a doença.

Recomenda-se cautela na generalização dos resultados, particularmente quanto à aparente ineficácia do preservativo, que pode refletir viés de memória ou subnotificação de práticas de risco. Portanto, futuros estudos devem incorporar desenhos longitudinais e amostras representativas de diferentes contextos socioespaciais.

## Data Availability

Os conjuntos de dados relacionados a este artigo estarão disponíveis mediante solicitação ao autor correspondente.

# Translation: Prueba rápida reactiva para sífilis entre jóvenes: prevalencia y factores asociados*

**DOI:** 10.1590/1518-8345.7488.4957

**Authors:** William Caracas Moreira, Leidyanny Barbosa de Medeiros, Rute Xavier Silva, Jordana de Almeida Nogueira, Mônica Oliveira Batista Oriá, Oriana Deyze Correia Paiva Leadebal

**Affiliations:** 1 Universidade Federal do Ceará, Campos Porangabuçu, Fortaleza, CE, Brasil.; 2 Universidade Federal da Paraíba, Campus I, João Pessoa, PB, Brasil.; 3 Becario de la Coordenação de Aperfeiçoamento de Pessoal de Nível Superior (CAPES), Brasil.; 4 Becaria del Conselho Nacional de Desenvolvimento Científico e Tecnológico (CNPq), Brasil.

**Keywords:** Sífilis, Adulto Joven, Conducta Sexual, Vulnerabilidad Sexual, Enfermedades de Transmisión Sexual, Prevalencia

## Abstract

**Objetivo:**

analizar la prevalencia de la prueba rápida reactiva para sífilis entre jóvenes y sus factores asociados.

**Método:**

estudio transversal, desarrollado en dos Centros de Pruebas y Asesoramiento. Involucró a 301 jóvenes, cuya información fue recolectada mediante un instrumento validado y analizada por estadística descriptiva, pruebas de chi-cuadrado, razón de prevalencia y regresión logística.

**Resultados:**

los jóvenes presentaron una prevalencia de 14,9% para la prueba rápida reactiva para sífilis, con asociaciones significativas con factores sociodemográficos (sexo masculino, identidad trans y orientación sexual no heterosexual), socioestructurales (bajos ingresos, vivienda independiente e historial de institucionalización) y conductuales (sexo transaccional, prácticas sexuales de riesgo, antecedente de infecciones de transmisión sexual y consumo de drogas). El análisis multivariado confirmó mayores probabilidades de prueba rápida reactiva para sífilis entre jóvenes con bajos ingresos, sexo masculino y antecedente de infecciones de transmisión sexual, lo que refuerza la vulnerabilidad de estos grupos.

**Conclusión:**

en suma, la prevalencia y los factores asociados a la prueba rápida reactiva para sífilis entre jóvenes atraviesan una estructura interseccional con múltiples capas de vulnerabilidades, contemplando dimensiones sociodemográficas, socioestructurales y conductuales.

## Destacados

**(1)** La prevalencia de la prueba rápida reactiva para sífilis entre jóvenes se estimó en 14,9%.

**(2)** El sexo masculino y las ITS previas aumentan el riesgo de prueba rápida reactiva para sífilis.

**(3)** Los factores socioestructurales amplían la vulnerabilidad a la prueba rápida reactiva para sífilis.

**(4)** Las prácticas sexuales de riesgo se asociaron con la prueba rápida reactiva para sífilis.

**(5)** Los bajos ingresos y la discriminación elevan la probabilidad de prueba rápida reactiva para sífilis.

## Introducción

La sífilis es una de las Infecciones de Transmisión Sexual (ITS) de notificación obligatoria, causada por *Treponema pallidum* y transmitida por: vía materno-fetal, durante la gestación; en el momento del parto, por vía sanguínea; o mediante relaciones sexuales sin el uso adecuado del preservativo^1^. Con diagnóstico accesible por el Sistema Único de Salud (SUS), la infección puede ser detectada y tratada rápidamente para evitar la evolución clínica, la aparición de complicaciones, el aumento de los casos de sífilis congénita y de nuevos casos de la infección adquirida^2^. Aun así, estudios recientes destacan el carácter reemergente y un número elevado de casos entre los jóvenes, en ascenso, especialmente entre aquellos menores de 24 años^1^ ^)-(^ ^3^.

En lo que se refiere a las estrategias de diagnóstico precoz, se dispone de la prueba rápida (PR) para sífilis^4^, la cual consiste en un método inmunocromatográfico capaz de detectar anticuerpos contra *Treponema pallidum* a partir de pequeñas muestras de sangre, obtenidas preferentemente por punción digital, lo que favorece una recolección ágil y sencilla, incluso en entornos con poca infraestructura laboral^4^ ^)-(^ ^5^. El resultado, disponible en pocos minutos, puede clasificarse en reactivo o no reactivo para sífilis. El resultado no reactivo implica el refuerzo de las medidas preventivas y la posible repetición de la PR en 30 días, en caso de sospecha clínica o riesgo reciente. Por otro lado, los resultados reactivos requieren confirmación mediante prueba no treponémica, como el *Venereal Disease Research Laboratory* (VDRL), a fin de distinguir la infección activa de la cicatriz serológica^5^.

La juventud, comprendida entre la adolescencia y la adultez joven, se sitúa entre los 15 y los 24 años, período en el que las personas experimentan descubrimientos en diferentes aspectos, los cuales, a su vez, moldean las relaciones sociales y contribuyen a los comportamientos, entre ellos, los sexuales^6^. En esta perspectiva, los estudios resaltan que la expresión sexual de las personas se ha manifestado cada vez más tempranamente y está comúnmente asociada a déficits de conocimiento sobre ITS y a dificultades de los padres y profesionales de la salud para abordar la temática en la población, conforme a las condiciones de vulnerabilidad para la sífilis entre jóvenes^6^ ^)-(^ ^7^.

A nivel mundial, el estudio *Global Burden of Disease* demostró que, aunque la sífilis no figura como la ITS más incidente en números absolutos, su tasa estandarizada por edad presentó crecimiento continuo entre 1990 y 2021, entre los cuales se destaca Brasil, que registró el mayor aumento de esa tasa entre los países analizados^8^.

En este contexto, se detalla que, entre 2011 y 2022, Brasil notificó más de 1,1 millones de casos de sífilis adquirida, de los cuales 36% ocurrieron en jóvenes. Entre 2020 y 2021, la tasa de detección nacional aumentó de 59,1 a 78,5 casos por 100 mil habitantes, tendencia observada también en la región Nordeste, cuyos índices pasaron de 29,9 a 47,3 casos por 100 mil habitantes. Paraíba, estado del nordeste brasileño, mostró un crecimiento similar al patrón regional, aunque sin superar la media nacional^2^. Además, cabe resaltar que los datos de notificaciones obligatorias de sífilis, en Paraíba, siguen la tendencia global^9^, es decir, disminuyeron desde el inicio de la pandemia de COVID-19, lo que ciertamente no refleja una reducción de la ocurrencia de la infección, sino fragilidad en el diagnóstico, subnotificación y, consecuentemente, ausencia de tratamiento oportuno^10^.

Este estudio, además de valorar la PR para sífilis en el contexto de la salud pública^5^, pone en evidencia la vulnerabilidad de un grupo etario específico (jóvenes de 15 a 24 años) frente a la sífilis, diferenciándose de los demás estudios que, mayoritariamente, abordan poblaciones generales, gestantes o grupos clave relacionados, sobre todo, a la identidad de género y/o a la orientación sexual, según una revisión sistemática de la literatura^11^.

Sumada a la necesidad científica de contribuir al cumplimiento de las metas de los Objetivos de Desarrollo Sostenible (ODS), de la Agenda 2030 - Organización de las Naciones Unidas (ONU)^12^, la producción de estudios de prevalencia de sífilis adquirida entre jóvenes encuentra sólida justificación tanto en el escenario epidemiológico global y brasileño^2^ ^),(^ ^8^ como en la dinámica de la investigación en salud^7^.

Ante lo expuesto, el objetivo de este estudio fue analizar la prevalencia de la PR reactiva para sífilis entre jóvenes y sus factores asociados.

## Método

### Tipo o diseño del estudio

Estudio transversal, desarrollado a partir de un recorte de la investigación primaria titulada “VIH en la población joven: subsidios para el enfrentamiento de la epidemia a partir del análisis de factores socioestructurales y conductuales”, reportado según las recomendaciones del *Strengthening the Reporting of OBservational studies in Epidemiology* (STROBE)^13^.

### Lugar del estudio

Desarrollado en dos Centros de Pruebas y Asesoramiento de dos municipios sede de las macrorregiones de salud I y II, del estado de Paraíba, Brasil. Estos servicios funcionan por demanda espontánea o por referencia de otros servicios, en los turnos matutino y vespertino, atienden a 134 de los 223 municipios del estado y ofrecen acciones de promoción, prevención, diagnóstico y tratamiento en salud sexual. 

### Período

La recolección de datos se llevó a cabo de febrero a julio de 2021.

### Población

La población estuvo compuesta por jóvenes de entre 15 y 24 años.

### Definición de la muestra

La muestra fue definida mediante cálculo para poblaciones finitas; se consideró la estimación media mensual de PR realizada en los servicios investigados (Sede de la Macrorregión I- N=500; Sede de la Macrorregión II- N=320); nivel de significación del 5%; índice de confianza (IC) del 95%; y un valor anticipado de Prevalencia (P) basado en la tasa de detección de casos de VIH/sida en el grupo etario establecido, con foco en la investigación original (Sede de la Macrorregión I con P = 23,0%; Sede de la Macrorregión II con P = 8,6%); lo que definió la muestra en 301 jóvenes, de los cuales 200 correspondieron a la Sede de la Macrorregión I y 101 a la Sede de la Macrorregión II.

### Criterios de selección

La selección de los participantes ocurrió por muestreo no probabilístico; se adoptó el criterio de conveniencia en función de las particularidades del lugar de estudio. Se incluyeron las personas con estado serológico para sífilis desconocido en el momento de la investigación primaria y que buscaron los Centros de Pruebas y Asesoramiento con el fin de realizarse la PR para alguna de las ITS. Además, fueron excluidos aquellos que presentaban alguna discapacidad que les imposibilitara responder al formulario.

### Instrumento utilizado y variables del estudio

El instrumento fue construido y validado por investigadores del Núcleo de Estudios en VIH, Salud y Sexualidad (NEHAS)^14^. Estructurado en 45 preguntas distribuidas en cuatro segmentos: 1 - Identificación de la entrevista; 2 - Variables sociodemográficas (edad, sexo, aspectos inherentes a la sexualidad, color autodeclarado, años de estudio); 3 - Variables socioestructurales (trabajo, situación actual de la relación, condiciones de vivienda, ingreso familiar - se consideró el salario mínimo de R$ 1.100,00 [vigente en 2021], antecedente de institucionalización, discriminación, tratamiento psiquiátrico y victimización por violencia sexual); y 4 - Variables conductuales (inicio de las relaciones sexuales, antecedente de ITS, consentimiento en las relaciones sexuales, aspectos relacionados con las prácticas sexuales, sexo transaccional, consumo de drogas)^14^.

### Recolección de datos

La recolección de datos se llevó a cabo en los turnos de mañana y tarde, de acuerdo con la disponibilidad de los investigadores, variando según los días de la semana, hasta alcanzar la muestra pretendida.

Conforme a lo establecido en los criterios de inclusión y exclusión, los jóvenes que buscaron los Centros de Pruebas y Asesoramiento fueron informados sobre el estudio e invitados a participar. Para la aceptación fue necesaria la firma del Consentimiento Informado o del Asentimiento Informado para menores de 18 años. Por consiguiente, participaron en una entrevista individual, en ambiente privado, orientada por el instrumento de recolección de datos^14^, con abordaje cara a cara por un investigador capacitado para conducirla. Cabe destacar que este proceso precedió a la realización de la PR.

### Tratamiento y análisis de los datos

Los datos de interés de este estudio fueron trasladados de la base de datos de la investigación original y organizados en una hoja de cálculo de *Microsoft Excel* ^*®*^ , para su compilación y recategorización mediante el programa estadístico *Statistical Package for the Social Sciences* (SPSS), versión 20.0.

Para el análisis de la caracterización de los participantes, se calcularon las frecuencias absolutas y relativas, y medidas de tendencia central y de dispersión.

Para el análisis de la prevalencia, se consideró el número de PR reactivas para sífilis, dividido por el total muestral (n= 301). Para el cálculo de chi-cuadrado (X²) de Pearson y razón de prevalencia (RP) se consideró la asociación del desenlace PR reactiva para sífilis y los factores asociados. Se resalta que, para estos análisis, se excluyeron los datos referentes a las categorías: “ignorado”; “no quiso responder”; y “no supo responder”.

Para el análisis multivariado, se optó por utilizar el modelo de regresión logística, adoptándose el *backward* a fin de seleccionar las variables que mejor explicaran el fenómeno en estudio, considerando el nivel de significación del 5% (p < 0,05)^15^.

### Aspectos éticos

Este estudio siguió los preceptos éticos de la investigación que involucra a seres humanos previstos en la Resolución n.° 466/12 del Consejo Nacional de Salud; fue aprobado por el Comité de Ética en Investigación del *Centro de Ciências da Saúde* de la *Universidade Federal da Paraíba* - Dictamen n.º 3.935.713 (26/03/2020), así como se condujeron los procedimientos necesarios para garantizar la protección de la información accesible al equipo de investigación y el anonimato con ocasión de la publicación de los resultados.

## Resultados

De los 382 jóvenes abordados por los investigadores durante la recolección de datos, 301 aceptaron participar. Los participantes tenían una edad media de 20,83 años (desviación estándar ± 2,3), cuya caracterización sociodemográfica indicó predominio del grupo etario de 20 a 24 años (212/70,4%), sexo masculino (155/51,5%), color autodeclarado no blanco (217/72,1%), personas cisgénero (291/96,6%), heterosexuales (175/58,1%), con más de ocho años de estudio (259/86%) y, entre quienes actualmente no estudian, se encontró un porcentaje de 86% (163).

En cuanto a los factores intrínsecos a la dimensión socioestructural, hubo predominio de jóvenes con relación estable (172/57,3%), ingreso familiar entre uno y dos salarios mínimos (111/37,2%), que no trabajan (166/55,1%) y comparten vivienda solo con familiares (152/50,5%). También reportaron victimización por discriminación relacionada con la orientación sexual (104/34,7%) y con la identidad de género (57/18,9%), principalmente en escuelas y universidades (86/82,7% y 11/19,6%, respectivamente). Además, se reportó violencia sexual (84/27,9%) - perpetrada principalmente por desconocidos (29/35%) -, antecedente de tratamiento psiquiátrico (61/20,3%) y de privación de libertad o de institucionalización (10/3,3%).

En el perfil conductual de los jóvenes, se observó el predominio de las siguientes características: inicio de las relaciones sexuales hasta los 15 años (153/50,8%) - práctica no consentida por una parte de los jóvenes (15/5%) -; utilizar el preservativo con una frecuencia de “a veces” (167/55,4%); practicar sexo oral (237/78,7%) y vaginal (214/71,1%); y consumo de bebidas alcohólicas (174/57,8%); de estos, una proporción refirió (68/39,5%) haber dejado de utilizar el preservativo cuando estaba bajo el efecto de bebidas alcohólicas; otra afirmó que no recuerda si utilizó o no el preservativo en ese tipo de ocasión (11/6,4%).

Hubo relatos de sexo transaccional (29/9,6%), prácticas de sexo anal insertivo (114/37,9%) y receptivo (124/41,2%), diagnóstico previo de ITS (63/20,9%), dificultad para adquirir (32/10,6%) o negociar el uso del preservativo (103/34,2%) y también se reportó consumo de drogas ilícitas (103/34,2%).

En cuanto a la prevalencia y asociaciones inherentes a los resultados, se identificó PR reactiva para sífilis en el 14,9% de los jóvenes y asociación con factores sociodemográficos, socioestructurales y conductuales.

En lo que respecta a los factores sociodemográficos, el desenlace de la PR reactiva para sífilis se asoció con las variables pertinentes al sexo, identidad de género y orientación sexual (Tabla 1). En esta dimensión (Tabla 1), el sexo masculino presentó una RP de 3,8 (IC95%: 1,9-7,5) veces más probabilidad para PR reactiva para sífilis, así como los jóvenes cuya identidad de género era hombre trans o mujer trans tuvieron, respectivamente, una RP de 8,0 (IC95%: 1,7-36,9) y 10,1 (IC95%: 4,4-23,1). Además, ser homosexual o pansexual mostró, respectivamente, una RP de 4,4 (IC95%: 2,3-8,4) y 4,8 (IC95%: 1,6-14,2), lo que indica mayor vulnerabilidad a la PR reactiva para sífilis en estos grupos.

**Tabla 1:** Prevalencia (P*), razón de prevalencia (RP^†^) y prueba de chi-cuadrado de asociación entre el desenlace de la prueba rápida reactiva para sífilis y factores sociodemográficos de los jóvenes (n = 301). João Pessoa, PB, Brasil, 2021

Variables		Prueba rápida reactiva para sífilis (N)		P* (%)	RP^†^ [IC^‡^ 95,0%]	X² p-valor^§^
		Sí	No			
Grupo etario	15-19 años	08	81	9,0	1	0,060
	20-24 años	37	175	17,5	1,9 [0,9; 4,0]	
Sexo	Femenino	09	137	6,2	1	0,001^||^
	Masculino	36	119	23,2	3,8 [1,9; 7,5]	
Color autodeclarado	Blanco	12	72	14,3	1	0,841
	No blanco	33	184	15,2	1,00 [0,6; 1,9]	
Años de estudio	≤ 8 años	04	38	9,5	1	0,288
	> 8 años	41	218	15,8	1,7 [0,6; 4,4]	
Estudia	Sí	19	119	13,8	1	0,597
	No	26	137	16,0	1,2 [0,6; 2,0]	
Identidad de género	Hombre	30	116	20,5	3,3 [1,6; 6,7]	0,001^||^
	Mujer	09	136	6,2	1	
	Hombre trans	01	1	50,0	8,0 [1,7; 36,9]	
	Mujer trans	05	3	62,5	10,1 [4,4; 23,1]	
Orientación sexual	Heterosexual	12	163	6,9	1	0,001^||^
	Homosexual	24	55	30,4	4,4 [2,3; 8,4]	
	Bisexual	06	32	15,8	2,0 [0,9; 5,7]	
	Pansexual	03	6	33,3	4,8 [1,6; 14,2]	

*P = Prevalencia; ^†^RP = Razón de prevalencia; ^‡^IC = Intervalo de Confianza; ^§^X² valor p = valor p de la prueba de chi-cuadrado para la comparación de proporciones; ^||^valor p <0,05 = Asociación entre la prueba rápida reactiva para sífilis y la variable correspondiente

En la dimensión comprendida por factores socioestructurales, se verificó la asociación estadística entre la PR reactiva para sífilis y las siguientes variables: ingreso familiar, con quien comparte la vivienda, victimización derivada de discriminación por identidad de género y orientación sexual, y haber estado preso(a) o institucionalizado(a). Así, se destaca que el ingreso familiar inferior a un salario mínimo elevó la RP a 2,3 (IC95%: 1,2-4,6) veces para PR reactiva para sífilis. Además, vivir solo con amigos o solo presentó, respectivamente, una RP de 3,9 (IC95%: 1,9-8,0) y 3,2 (IC95%: 1,6-6,3). Fenómeno semejante ocurre con jóvenes que tenían antecedente de prisión/institucionalización, con una RP de 2,8 (IC95%: 1,7-6,4) (Tabla 2).

**Tabla 2:** Prevalencia (P*), razón de prevalencia (RP^†^) y prueba de chi-cuadrado de asociación entre el desenlace de la prueba rápida reactiva para sífilis y factores socioestructurales de los jóvenes (n = 301). João Pessoa, PB, Brasil, 2021

Variables		Prueba rápida reactiva para sífilis (N)		P* (%)	RP^†^ [IC^‡^ 95,0%]	X² p valor^§^
		Sí	No			
Ingreso familiar	≤ 1 SM^||^	23	70	24,7	2,3 [1,2; 4,6]	0,008^¶^
	1 a 2 SM^||^	12	99	10,8	1,0 [0,5; 2,2]	
	> 2 SM^||^	10	84	10,6	1	
Trabaja	Sí	20	115	14,8	1	0,953
	No	25	141	15,1	1,0 [0,6; 1,7]	
Con quién comparte la vivienda	Solo con amigos	08	13	38,1	3,9 [1,9; 8,0]	0,001^¶^
	Solo (a)	12	26	31,6	3,2 [1,6; 6,3]	
	Solo con la pareja	07	37	15,9	1,6 [0,7; 3,7]	
	Solo con familiares	15	137	9,9	1	
	Pareja y amigos o familiares	03	43	6,5	0,7 [0,2; 2,2]	
Situación de la relación	Fijo	17	155	9,9	1	0,051
	Casual	11	33	25,0	2,5 [1,3; 5,0]	
	Con más de una pareja	02	12	14,3	1,4 [0,3; 5,6]	
	Sin relación	15	55	21,4	2,1 [1,1; 4,1]	
Discriminación por identidad de género	Sí	15	42	26,3	2,1 [1,2; 3,7]	0,008^¶^
	No	30	214	12,3	1	
Discriminación por orientación sexual	Sí	29	75	27,9	3,4 [1,9; 6,0]	0,001^¶^
	No	16	179	8,2	1	
Victimización por violencia sexual	Sí	16	68	19,0	1,4 [0,8; 2,5]	0,215
	No	29	188	13,4	1	
Antecedente de encarcelamiento o institucionalización	Sí	04	6	40,0	2,8 [1,7; 6,4]	0,024^¶^
	No	41	250	14,1	1	
Antecedente de tratamiento psiquiátrico	Sí	11	50	18,0	1,3 [0,7; 2,3]	0,450
	No	34	206	14,2	1	

*P = Prevalencia; ^†^RP = Razón de prevalencia; ^‡^IC = Intervalo de Confianza; ^§^X² valor p = valor p de la prueba de chi-cuadrado para la comparación de proporciones; ^||^SM = Salario mínimo de R$ 1.100,00, Brasil, vigente en 2021; ^¶^valor p <0,05 = Asociación entre la prueba rápida reactiva para sífilis y la variable correspondiente

En cuanto a los factores conductuales, la PR reactiva para sífilis se asoció con la falta de consentimiento en la primera relación sexual, la frecuencia de uso del preservativo, el sexo transaccional, las prácticas de sexo vaginal, anal insertivo y receptivo, el antecedente de ITS y el consumo de drogas ilícitas (Tabla 3). Así, se observa que la falta de consentimiento en la primera relación sexual aumentó la RP en 2,3 (IC95%: 1,1-5,1) para la PR reactiva para sífilis, al igual que las prácticas sexuales: sexo transaccional con RP de 3,8 (IC95%: 2,2-6,4), sexo anal insertivo con RP de 2,6 (IC95%: 1,5-4,6) y receptivo con RP de 1,7 (IC95%: 1,1-3,0). Además, los jóvenes que no practicaban sexo vaginal presentaron una RP 3,8 (IC95%: 2,2-6,6) veces más probabilidad para PR reactiva para sífilis (Tabla 3).

**Tabla 3:** Prevalencia (P*), razón de prevalencia (RP^†^) y prueba de chi-cuadrado de asociación entre el desenlace de la prueba rápida reactiva para sífilis y factores conductuales de los jóvenes (n = 301). João Pessoa, PB, Brasil, 2021

Variables		Prueba rápida reactiva para sífilis (N)		P* (%)	RP^†^ [IC^‡^ 95,0%]	X² p-valor^§^
		Sí	No			
Coitarca (n= 294)	≤ 15 años	25	128	16,3	1,1 [0,6; 2,0]	0,608
	> 15 años	20	121	14,2	1	
Primera relación sexual consentida (n= 298)	Sí	40	243	14,1	1	0,043^||^
	No	05	10	33,3	2,3 [1,1; 5,1]	
Dificultad para adquirir preservativos (n= 299)	Sí	08	24	25,0	1,8 [0,9; 3,5]	0,096
	No	37	230	13,9	1	
Frecuencia de uso del preservativo (n= 297)	Siempre	21	40	34,4	5,9 [2,1; 16,3]	0,001^||^
	A veces	20	147	12,0	2,0 [0,7; 5,8]	
	Nunca	04	65	5,8	1	
Dificultad para negociar el uso del preservativo (n= 294)	Siempre	03	28	9,7	1	0,265
	A veces	15	57	20,8	2,1 [0,6; 6,9]	
	Nunca	27	164	14,1	1,4 [0,4; 4,5]	
Sexo transaccional	Sí	13	16	44,8	3,8 [2,2; 6,4]	0,001^||^
	No	32	240	11,8	1	
Practica sexo vaginal (n=297)	Sí	18	196	8,4	1	0,001^||^
	No	27	56	32,5	3,8 [2,2; 6,6]	
Practica sexo oral (n=297)	Sí	38	199	16	1,3 [0,6; 2,9]	0,399
	No	7	53	11,7	1	
Practica sexo anal insertivo (n=297)	Sí	28	86	24,6	2,6 [1,5; 4,6]	0,001^||^
	No	17	166	9,3	1	
Practica sexo anal receptivo (n=297)	Sí	25	99	20,2	1,7 [1,1; 3,0]	0,041^||^
	No	20	153	11,6	1	
Antecedente de ITS^¶^ (n= 289)	Sí	30	33	47,6	7,1 [4,1; 12,5]	0,001^||^
	No	15	211	6,6	1	
Consume bebida alcohólica	Sí	31	143	17,8	1,6 [0,9; 2,9]	0,103
	No	14	113	11,0	1	
Consume drogas ilícitas	Sí	23	80	22,3	2,0 [1,2; 3,4]	0,010^||^
	No	22	176	11,1	1	

*P = Prevalencia; ^‡^IC = Intervalo de Confianza; ^§^X² valor p = valor p de la prueba de chi-cuadrado para la comparación de proporciones; ^||^valor p <0,05 = Asociación entre la prueba rápida reactiva para sífilis y la variable correspondiente; ^¶^ITS = Infecciones de Transmisión Sexual

Asimismo, el antecedente previo de ITS y el consumo de drogas ilícitas aumentaron la RP en, respectivamente, 7,1 (IC95%: 4,1-12,5) y 2,0 (IC95%: 1,2-3,4) para PR reactiva para sífilis. En cuanto a la frecuencia de uso del preservativo, aquellos que respondieron utilizarlo siempre presentaron una RP 5,9 (IC 2,1-16,3) veces más probabilidad para la PR reactiva para sífilis en comparación con los jóvenes que respondieron utilizarlo a veces o nunca (Tabla 3).

Con base en el análisis por regresión logística, ser un joven de 15 a 19 años constituye un efecto protector para la PR reactiva para sífilis en comparación con los jóvenes de 20 a 24 años. Sin embargo, aquellos con ingreso inferior a un salario mínimo o entre uno y dos salarios mínimos presentaron 2,9 y 2,4 veces más probabilidades de PR reactiva para sífilis, respectivamente, en comparación con los jóvenes con ingreso superior a tres salarios mínimos. De igual manera, ser de sexo masculino aumentó tres veces las probabilidades de PR reactiva para sífilis, en comparación con el sexo femenino, y tener antecedente de alguna ITS representó 8,4 veces más probabilidades de PR reactiva para sífilis, en relación con quienes no relataron tal antecedente (Tabla 4).

**Tabla 4:** Modelo de regresión logística que contempla factores asociados a la prueba rápida reactiva para sífilis entre jóvenes (n = 301). João Pessoa, PB, Brasil, 2021

Variables	Coeficiente	Error estándar	Odds ratio	Intervalo de confianza (95%)	p valor*
Grupo etario de 15-19 años	-0,78	0,34	0,45	[0,23 - 0,89]	0,02^†^
Sexo masculino	1,11	0,43	3,06	[1,31 - 7,11]	0,01^†^
Ingreso familiar ≥ 3 SM^‡^					0,01^†^
Ingreso familiar ≤ 1 SM^‡^	1,09	0,42	2,98	[1,31 - 6,80]	0,01^†^
Ingreso familiar entre 1-2 SM^‡^	0,90	0,46	2,47	[1,01 - 6,09]	0,04^†^
Tener diagnóstico previo de ITS^§^	2,13	0,36	8,46	[4,11 - 17,42]	0,01^†^
**R² (Nagelkerke)** ^||^					70,5%
**Prueba de Hosmer-Lemeshow (p-valor)**					0,20

* *p* -valor = *p* valor del modelo de regresión logística; ^†^p<0,05 = Asociación entre la prueba rápida reactiva para sífilis y los factores investigados; ^‡^SM = Salario mínimo vigente = R$ 1.100,00, Brasil, 2021; ^§^ITS = Infección de Transmisión Sexual; ^||^R² (Nagelkerke) = Mide el desempeño del modelo a partir de las variables utilizadas

## Discusión

El perfil sociodemográfico presentado en este estudio se asemeja a la literatura nacional^16^ ^)-(^ ^17^ e internacional^7^ ^),(^ ^18^ ^)-(^ ^23^ relacionada con la prevalencia de jóvenes de sexo masculino^16^ ^)-(^ ^19^ ^),(^ ^21^ ^)-(^ ^22^, cisgénero^7^, heterosexuales^7^ ^),(^ ^16^, del grupo etario de 20 a 24 años^20^, no blancos^16^ ^),(^ ^19^ ^),(^ ^23^ y con más de ocho años de estudio en sus muestras^16^. Solo discrepó la variable “estudia actualmente”, pues la mayoría de las investigaciones que focalizan a los jóvenes tienden a abordar el escenario universitario, lo que consecuentemente influyó en la variable en cuestión^24^.

Los jóvenes de sexo masculino asumen el mayor riesgo no solo de PR reactiva para sífilis, sino también de otras ITS^21^ ^)-(^ ^22^; dicho esto, se explica que la vulnerabilidad puede estar vinculada a comportamientos como la búsqueda de parejas sexuales por internet, inicio precoz de las relaciones sexuales, mayor frecuencia de relaciones sexuales y baja adopción de prácticas de autocuidado y preservación de la salud sexual, en comparación con el sexo femenino^17^ ^)-(^ ^18^ ^),(^ ^21^ ^)-(^ ^22^.

Los jóvenes de 20 a 24 años presentan mayor vulnerabilidad a la PR reactiva para sífilis y otras ITS, especialmente las de sexo femenino, debido a la convergencia de factores sociodemográficos y económicos desfavorables, prácticas sexuales transaccionales y uso inconsistente de preservativos^25^ ^)-(^ ^26^. En este escenario, la persistencia de la desinformación sobre salud sexual y la exposición a comportamientos de riesgo amplifican esta vulnerabilidad, por lo que resulta fundamental el acceso a información adecuada y el análisis colectivo de los factores que interfieren en este proceso^27^.

Más allá de los factores individuales, los análisis bivariados y multivariados presentados en este estudio sugieren la vulnerabilidad de grupos poblacionales vinculados al medio LGBTQIAPN+, ya que señalan un mayor riesgo de PR reactiva para sífilis en personas de sexo masculino, transexuales, homosexuales o pansexuales. Esta coyuntura sociodemográfica puede estar relacionada con la desinformación provocada por una cascada de acontecimientos sustentados en el prejuicio estructural e histórico experimentado por este grupo, cuyos impactos se perciben en el acceso a los servicios de salud, en la disponibilidad de recursos e información para prevenir comportamientos sexuales de riesgo e ITS, y promover la salud sexual^28^ ^)-(^ ^30^.

Asimismo, las variables socioestructurales reflejan patrones complejos de vulnerabilidad en este grupo poblacional, con destaque para las interseccionalidades entre condiciones económicas, situaciones de violencia y salud mental^31^. Estas condiciones se encuentran consolidadas en la literatura cuando se visualizan de forma aislada^32^; sin embargo, la evidencia de esta coyuntura, con sus interseccionalidades, en estudios de prevalencia vinculados al riesgo de PR reactiva para sífilis aún ha sido poco explorada.

En este contexto, a pesar del potencial de transformación social de los entornos educativos en cuestiones de respeto y dignidad humana, las escuelas y universidades perpetúan una estructura prejuiciosa, lo que configura espacios de reproducción de violencias LGBTfóbicas^29^ ^),(^ ^33^. Otro factor intrínseco a la vulnerabilidad socioestructural de estos jóvenes está relacionado con el antecedente de privación de libertad, particularmente relevante en poblaciones jóvenes periféricas, que afecta la forma en que los jóvenes se relacionan (al asumir comportamientos sexuales de riesgo), su inserción en el mercado laboral y la manera en que perciben el acceso a la salud^34^.

Asimismo, el relato de violencia sexual en adolescentes y jóvenes está presente desde grandes centros urbanos hasta comunidades rurales^35^, cuya predominancia de perpetradores desconocidos, señalada en este estudio, contrasta parcialmente con un estudio realizado en el interior de Piauí, que indicó a las parejas íntimas y amigos como los principales perpetradores, lo que sugiere que se trata de la realidad de contextos específicos de violencia urbana o de subnotificación de casos intrafamiliares^36^.

El ingreso familiar es un factor socioestructural inherente al acceso a la salud y a la información, puesto que, cuanto mayor es el ingreso, mejor es el acceso a los servicios y acciones de salud^37^. Dicho esto, los estudios demuestran que, cuanto menor es el ingreso familiar, mayor es la probabilidad de uso inconsistente de preservativos y, consecuentemente, mayor el riesgo de ITS^31^ ^),(^ ^37^ ^)-(^ ^38^.

En cuanto a las variables “no trabaja actualmente” y “no estudia actualmente”, la literatura esclarece que los jóvenes vivencian aspectos típicos de la adultez emergente, permeada por marcadores sociales como género, escolaridad y experiencia laboral, además de verse afectados por la precariedad del mercado de trabajo^23^. Esta condición, además de limitar oportunidades de desarrollo personal y social, expone a estos jóvenes a múltiples situaciones de vulnerabilidad. Tales factores están asociados a contextos de mayor aislamiento social, dificultad de acceso a la información y a los servicios de salud, y adopción de comportamientos de riesgo, lo que hace que esta población joven sea especialmente susceptible a las ITS^23^.

En nuestra muestra, al menos la mitad de los jóvenes refirieron vivir solo con familiares, lo que, con base en la literatura, constituye un factor de protección frente a las ITS^39^ ^)-(^ ^40^. Sin embargo, aquellos cuya vivienda es con amigos y/o solos representaron mayor riesgo de PR reactiva para sífilis y para otras ITS, ya que el joven posee mayor libertad y poca supervisión, asumiendo, por tanto, conductas sexuales de riesgo^39^. Además, se destaca que el grupo etario de 15 a 19 años, entendido como factor de protección frente a la PR reactiva para sífilis en este estudio, puede explicarse por la convivencia con familiares, la alta supervisión y el inicio tardío de las relaciones sexuales^40^.

En relación con los factores conductuales, se observa que el contexto relacional de los jóvenes determina patrones de comportamiento que influyen directamente en las dinámicas de protección sexual y crea escenarios de vulnerabilidad epidemiológica diferenciados^24^. Sin embargo, el riesgo estimado por el estado de la relación asumido por los jóvenes frente a las ITS aún no es un consenso científico. Dicho esto, los análisis conductuales demuestran que las parejas ocasionales presentan mayor propensión a la negligencia profiláctica, ya sea por olvido o por despreocupación deliberada, mientras que las relaciones estables favorecen la sustitución progresiva del preservativo por criterios de confianza interpersonal^21^ ^),(^ ^24^ ^),(^ ^41^. En este sentido, ambos modelos pueden aumentar la exposición a la sífilis y a otras ITS, ya que la reducción en el uso consistente de barreras físicas facilita la transmisión durante las prácticas sexuales, independientemente de la percepción subjetiva de seguridad^24^.

Una revisión de alcance mostró que la iniciación sexual entre jóvenes ocurre, en promedio, a los 15 años^24^. En este contexto, la primera experiencia sexual asume relevancia epidemiológica, pues la ausencia de preservativo y/o la falta de consentimiento potencian los comportamientos de riesgo y aumentan la vulnerabilidad a las ITS^21^ ^),(^ ^24^. Así, el inicio de las relaciones sexuales desprotegido o no consensuado configura un importante factor para la exposición y perpetuación de estas infecciones entre los jóvenes^21^ ^),(^ ^42^.

En lo que respecta a la dimensión del conocimiento anteriormente mencionada, se percibe que la coerción y el abuso sexual no están relacionados únicamente con el embarazo no deseado, sino también con el riesgo de ITS. A su vez, la incidencia de relaciones sexuales no consensuadas es un indicador de las desigualdades de género y de los patrones de masculinidad predominantes^31^. Así, se observa que la iniciación sexual coaccionada y forzada está asociada al uso inconsistente de preservativos, a experiencias recientes de violencia y a resultados de salud mental entre jóvenes, coyuntura que explicita las múltiples capas de vulnerabilidad de los jóvenes, especialmente de las personas de sexo femenino^31^ ^),(^ ^42^. 

En la actualidad, se reconoce que la práctica sexual transaccional se traduce en un factor de vulnerabilidad conductual, con interfaces sociales, económicas y/o estructurales^21^ ^),(^ ^24^. En este sentido, un estudio señaló que los jóvenes en situación de calle frecuentemente descuidan el uso de preservativos durante las relaciones sexuales transaccionales debido a presiones de parejas íntimas, necesidades financieras inmediatas y miedo a la violencia; esto describe un escenario propicio para la transmisión de la sífilis y de otras ITS^43^. 

La dinámica anteriormente mencionada se reproduce en diferentes contextos: entre universitarios sudafricanos, el 1,4% de los 918 investigados refirió involucrarse en esta práctica^44^; mientras que, en adolescentes estadounidenses de enseñanza secundaria, la prevalencia alcanzó el 7,4%, con destaque para el sexo masculino (2,5 veces más propenso)^45^. Adicionalmente, factores estructurales como la pobreza, la exclusión social y el acceso limitado a servicios de salud perpetúan ciclos de vulnerabilidad y dificultan tanto la prevención como el diagnóstico precoz de estas infecciones^24^ ^),(^ ^43^.

Entre las prácticas sexuales asumidas por los jóvenes, el sexo anal emerge como factor de mayor predisposición a la PR reactiva para sífilis y a otras ITS^24^, por la combinación de aspectos como: mayor fragilidad del epitelio rectal frente a infecciones; menor percepción de riesgo, cuyo sentimiento de invulnerabilidad posibilita la adopción de comportamientos sexuales de riesgo; y déficits en el acceso a información adecuada y a servicios de salud^46^.

Está consolidado en la literatura que el antecedente previo de ITS indica exposición anterior a comportamientos de riesgo, los cuales tienden a repetirse a lo largo del tiempo^21^ ^),(^ ^24^. Revisiones de la literatura demuestran una asociación significativa entre antecedentes de ITS y mayor riesgo de PR reactiva para sífilis y otras infecciones, corroborando la persistencia de vulnerabilidades conductuales^21^ ^),(^ ^24^. En este sentido, el fenómeno se detalla en estudios que evidenciaron que las personas con antecedente de ITS presentan un riesgo 1,8 veces más probabilidad de adquirir otras ITS^27^, mientras que el diagnóstico previo de otras dos ITS eleva en 5,83 veces la probabilidad de infección por sífilis^47^.

En otro contexto, la asociación entre el consumo de alcohol y/o de drogas ilícitas y las prácticas sexuales inseguras entre jóvenes configura un eje crítico para la transmisión de sífilis y otras ITS. Un estudio señaló que el uso de sustancias psicoactivas altera los patrones de juicio, reduce la percepción de riesgo durante las interacciones sociales; en eventos festivos, los jóvenes se encuentran menos preocupados por los riesgos subsiguientes y, en consecuencia, asumen comportamientos sexuales de riesgo^48^.

La frecuencia de uso del preservativo representa un factor central en la prevención de las ITS, dado que es ampliamente reconocido que la práctica sexual desprotegida aumenta el riesgo de adquisición de estas infecciones^2^. No obstante, los resultados de este estudio divergen de la literatura al sugerir que los jóvenes que utilizan preservativo con mayor regularidad, contradictoriamente, presentan mayor probabilidad de PR reactiva para sífilis. Tal fenómeno encuentra precedente en una investigación internacional, a partir de una cohorte estadounidense que asoció el uso frecuente de preservativos con la reactividad de pruebas para sífilis^25^. Estos hallazgos exigen investigaciones adicionales para esclarecer potenciales factores de confusión; debe considerarse el consenso científico sobre la eficacia del preservativo como método preventivo y la posible influencia de sesgos de información derivados de subnotificación u omisión por parte de los participantes.

Con base en las evidencias presentadas, las políticas públicas dirigidas a la prevención de las ITS y a la promoción de la salud sexual entre jóvenes deben priorizar intervenciones multinivel que combinen educación en salud, estrategias comunitarias y el uso de tecnologías digitales^49^ ^)-(^ ^50^. En este contexto, la experiencia del *Impact Triad* ^*®*^ demuestra que los abordajes basados en navegación entre pares, que utilizan líderes comunitarios capacitados para difundir información y facilitar el acceso a los servicios de salud, aumentan significativamente la realización de PR para ITS, el uso de preservativos y la adhesión a la profilaxis preexposición, además de fortalecer el apoyo social y reducir la discriminación entre jóvenes de grupos minoritarios^50^.

Paralelamente, una revisión sistemática evidencia que las plataformas de salud móvil (*mHealth*), como mensajes de texto, llamadas de voz y aplicaciones interactivas, son bien aceptadas por los jóvenes por ofrecer privacidad, conveniencia y lenguaje adaptado. Por lo tanto, adicionalmente, se recomienda que las políticas públicas integren acciones presenciales y digitales, promuevan la participación activa de los jóvenes en la elaboración de las estrategias, capaciten a profesionales de la salud y comunidades para abordar temas sensibles, e inviertan en campañas educativas continuas e intersectoriales, a fin de posibilitar el acceso universal y equitativo a la prevención y al cuidado en salud sexual^49^.

En la práctica de enfermería y salud, los hallazgos demandan cribado prioritario para grupos clave entre los jóvenes, intervenciones educativas basadas en tecnologías digitales e integración de evaluaciones psicosociales en los protocolos de enfermería. La actuación multidisciplinaria debe combinar PR (enfermería y demás profesionales de la salud debidamente capacitados para ello), articulación de redes de protección (asistencia social) y manejo del trauma (psicología), con foco en reducir inequidades en el acceso a la salud y maximizar la promoción de la salud sexual.

A pesar de las limitaciones de este estudio, como el estudio primario se centró exclusivamente en el VIH, sus análisis se limitaron a las variables relacionadas con esta infección. Esta delimitación metodológica resultó en la ausencia de datos específicos del VDRL, examen crucial para la cuantificación de la carga serológica de la sífilis. Se asume la posibilidad de sesgo de selección de los participantes, puesto que se trata de jóvenes que buscaron atención y ya conocen los Centros de Pruebas y Asesoramiento. Además, el muestreo por conveniencia en centros urbanos puede subestimar la realidad de los jóvenes de comunidades rurales.

## Conclusión

La prevalencia de PR reactiva para sífilis en jóvenes se estimó en 14,9%, cuyo contexto señala una estructura interseccional permeada por múltiples capas de vulnerabilidades que asocia el fenómeno con factores sociodemográficos (sexo masculino, ser hombre o mujer trans y homosexual o pansexual), socioestructurales (bajo ingreso familiar, vivienda independiente y antecedente de institucionalización) y conductuales (primera relación sexual no consentida, sexo transaccional, prácticas sexuales de riesgo, antecedente de ITS y consumo de drogas).

Además, en lo que se refiere a la PR reactiva para sífilis entre jóvenes, el modelo de regresión logística mostró que, mientras que el grupo etario de 15 a 19 años se comportó como factor protector en comparación con el grupo de 20 a 24 años, el bajo ingreso (inferior a dos salarios mínimos) y el sexo masculino aumentaron las probabilidades de PR reactiva. Notablemente, el antecedente previo de ITS se destacó como un importante factor de riesgo, elevando 8,4 veces las probabilidades de PR reactiva para la enfermedad.

Se recomienda cautela en la generalización de los resultados, particularmente en cuanto a la aparente falta de efecto protector del preservativo en los resultados, que puede reflejar sesgo de memoria o subnotificación de prácticas de riesgo. Por lo tanto, futuros estudios deben incorporar diseños longitudinales y muestras representativas de diferentes contextos socioespaciales.

## Data Availability

Los conjuntos de datos relacionados con este artículo estarán disponibles previa solicitud al autor correspondiente.
